# Supramolecular frameworks based on [60]fullerene hexakisadducts

**DOI:** 10.3762/bjoc.13.1

**Published:** 2017-01-02

**Authors:** Andreas Kraft, Johannes Stangl, Ana-Maria Krause, Klaus Müller-Buschbaum, Florian Beuerle

**Affiliations:** 1 Institut für Organische Chemie & Center for Nanosystems Chemistry, Universität Würzburg, Am Hubland, 97074 Würzburg, Germany; 2 Institut für Anorganische Chemie, Universität Würzburg, Am Hubland, 97074 Würzburg, Germany

**Keywords:** fullerenes, hexakisadducts, hydrogen bonding, porous materials, structure elucidation

## Abstract

[60]Fullerene hexakisadducts possessing 12 carboxylic acid side chains form crystalline hydrogen-bonding frameworks in the solid state. Depending on the length of the linker between the reactive sites and the malonate units, the distance of the [60]fullerene nodes and thereby the spacing of the frameworks can be controlled and for the most elongated derivative, continuous channels are obtained within the structure. Stability, structural integrity and porosity of the material were investigated by powder X-ray diffraction, thermogravimetry and sorption measurements.

## Introduction

**Figure 1:**
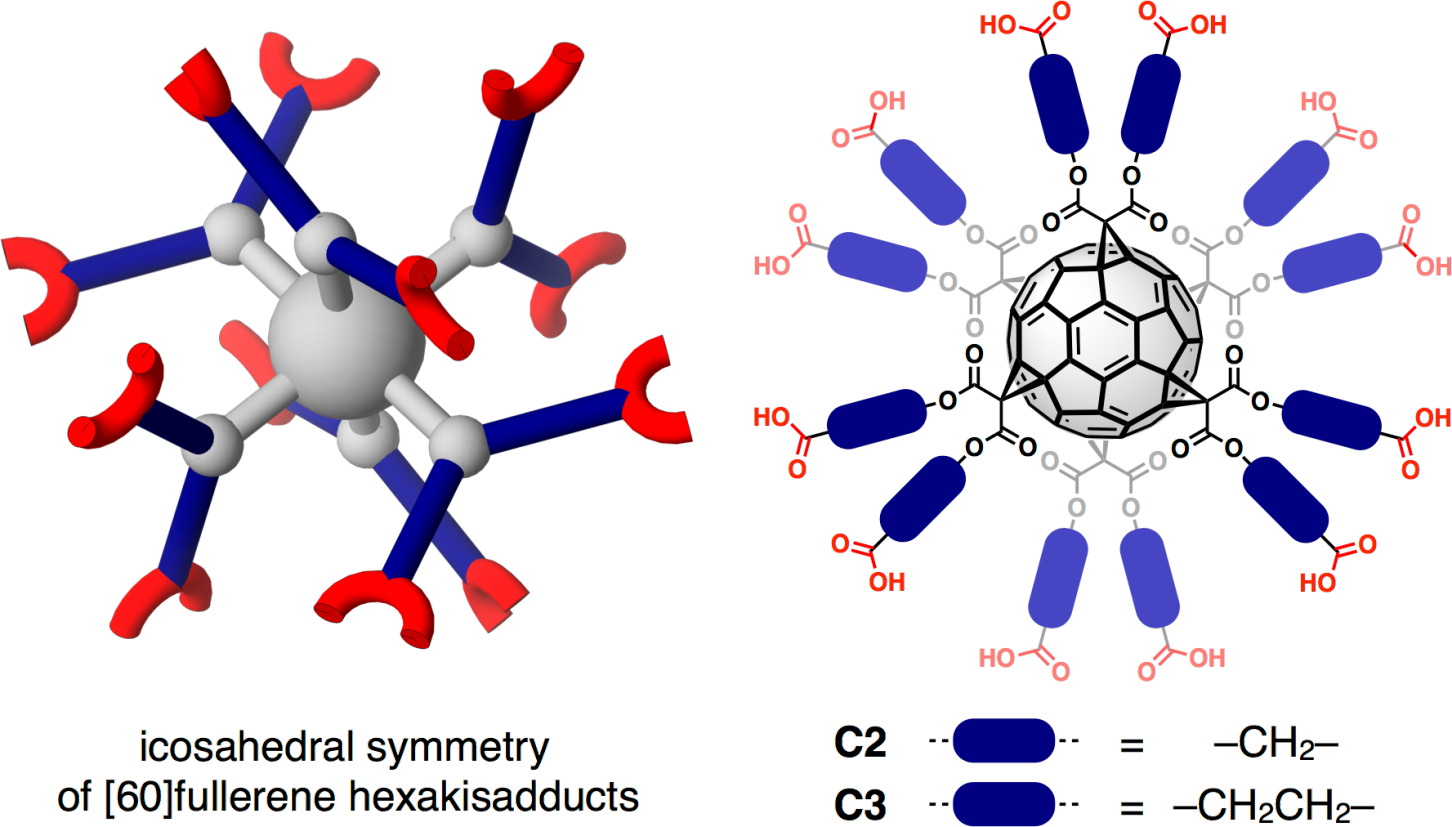
Icosahedral arrangement of functional addends for [60]fullerene hexakisadducts with dodecaacids **C2** and **C3** as prototypical examples for such carbon nanostructures.

The utilization of confined nanospace in rigid frameworks [1], which are derived from small molecular precursors under dynamic conditions, has emerged as a novel design paradigm for functional materials with the prospect of applications in gas storage [2–3], catalysis [4–5], luminescence [6–9] and sensing [10–13] or optoelectronics [14–16]. Owing to a modular approach, building blocks and cross-linking interactions can be varied over a wide range resulting in metal-organic frameworks (MOFs) [17–18], covalent organic frameworks (COFs) [19–20] or covalent organic cage compounds [21–28] as the most prominent examples for such artificial porous materials. Purely organic systems such as COFs usually benefit from very low densities, high thermal stabilities and metal-free synthesis, but in most cases, have the disadvantages of poor crystallinity and limited processability or solution-phase characterization. In contrast, the formation of robust porous structures by means of supramolecular interactions between rigid organic molecules might be a promising alternative thus combining low-weight materials with easy processing. However, the crystallization of stable organic structures possessing permanent porosity is still quite challenging and only a limited number of examples for supramolecular crystals based on hydrogen bonding [29–45] or π–π-stacking [46] that retain porosity in the solid state under activation conditions have been reported so far. One possible way to enhance stability and shape-persistency might be the implementation of polyfunctional building blocks in order to strengthen the non-covalent interactions in a cooperative manner. In this regard, [60]fullerene hexakisadducts [47], which can arrange up to twelve functional sites with icosahedral symmetry, exhibit one of the highest degrees of functionalization for organic molecules (see Figure 1). In recent years, a variety of derivatives have been synthesized as spherical branching units [48–53] and, more recently, functionalized fullerene derivatives have been implemented into coordination compounds [54–56]. However, to the best of our knowledge, no fullerene-containing crystalline frameworks retaining permanent porosity in the solvent-free state have been reported so far.

Here, we report on the crystallization of three-dimensional hydrogen-bonding frameworks based on [60]fullerene hexakisadducts bearing twelve carboxylic acid groups icosahedrally arranged on the fullerene surface. By varying the spacer length, the solvent-filled pore systems in the solid-state structures have been tuned and structural features such as porosity of the materials have been investigated by PXRD, TGA analysis and sorption studies.

## Results and Discussion

**Figure 2:**
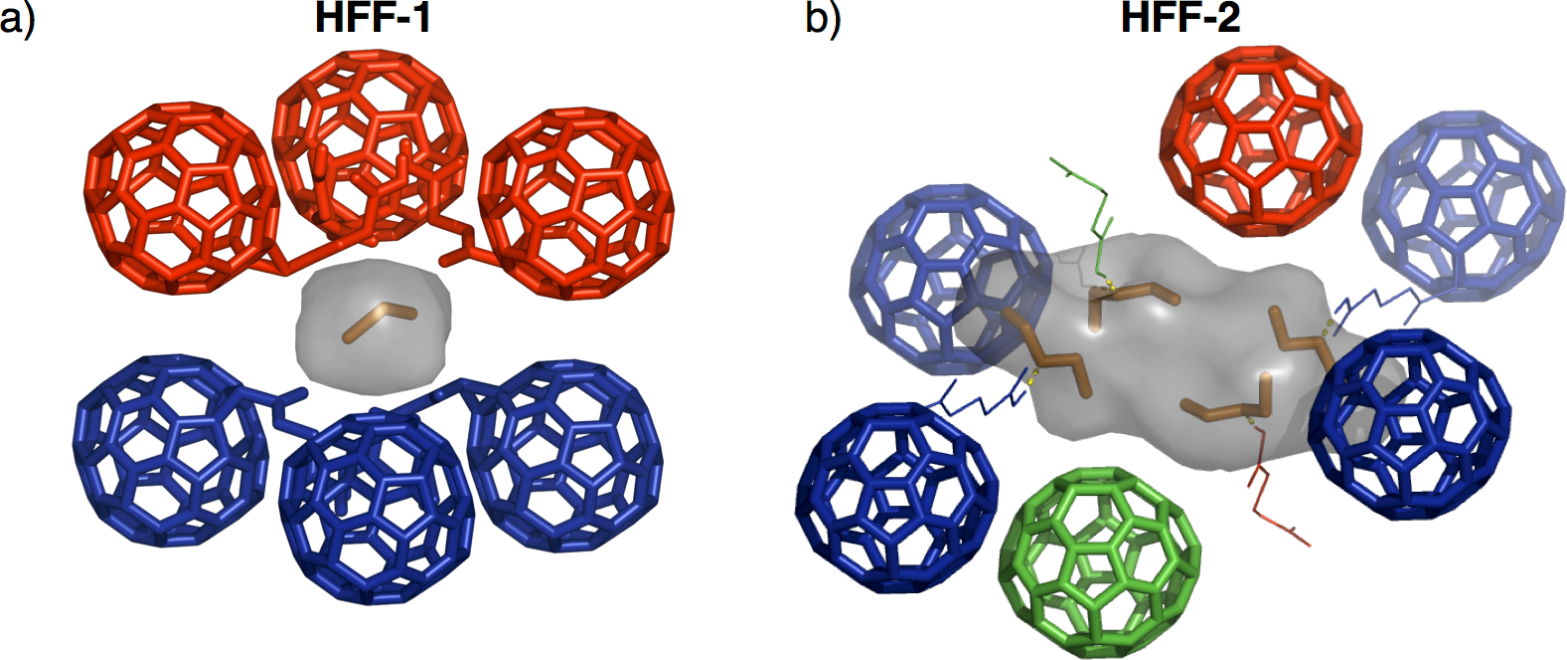
a) Small cavities within the octahedral sites of **HFF-1** filled with one CH_2_Cl_2_ molecule [57]; b) isolated cavities containing four Et_2_O molecules for **HFF-2** (void spaces are indicated as grey surfaces and most side arms are omitted for clarity, images are created with PyMOL [58]).

Recently, we reported on the synthesis and solid-state structure of dodecaacid **C2** (**C*n*** stands for *T*_h_ symmetrical hexakisadducts C_60_{C[COO(CH_2_)*_n_*_−1_COOH]_2_}_6_, see Figure 1) revealing a complex hydrogen-bonding network in the crystalline state [57]. Based on this initial finding, we also utilized **C2** and elongated derivative **C3** as organic connectivity centers in metal-organic assemblies obtained after reaction with Zn^2+^ ions [55], however, no crystal structure of metal-free **C3** has been reported yet. As a general packing motif for all fullerene-containing frameworks, the individual carbon building blocks are arranged in face centered cubic (fcc) packing with the distances between the molecules depending on the spacer length and the mode of cross-linking. For **HFF-1** (hydrogen-bonded fullerene framework) derived from **C2**, a densely packed structure is observed possessing only very small cavities within the octahedral sites of the fcc packing filled with one CH_2_Cl_2_ molecule (Figure 2a) [57].

**Scheme 1:**
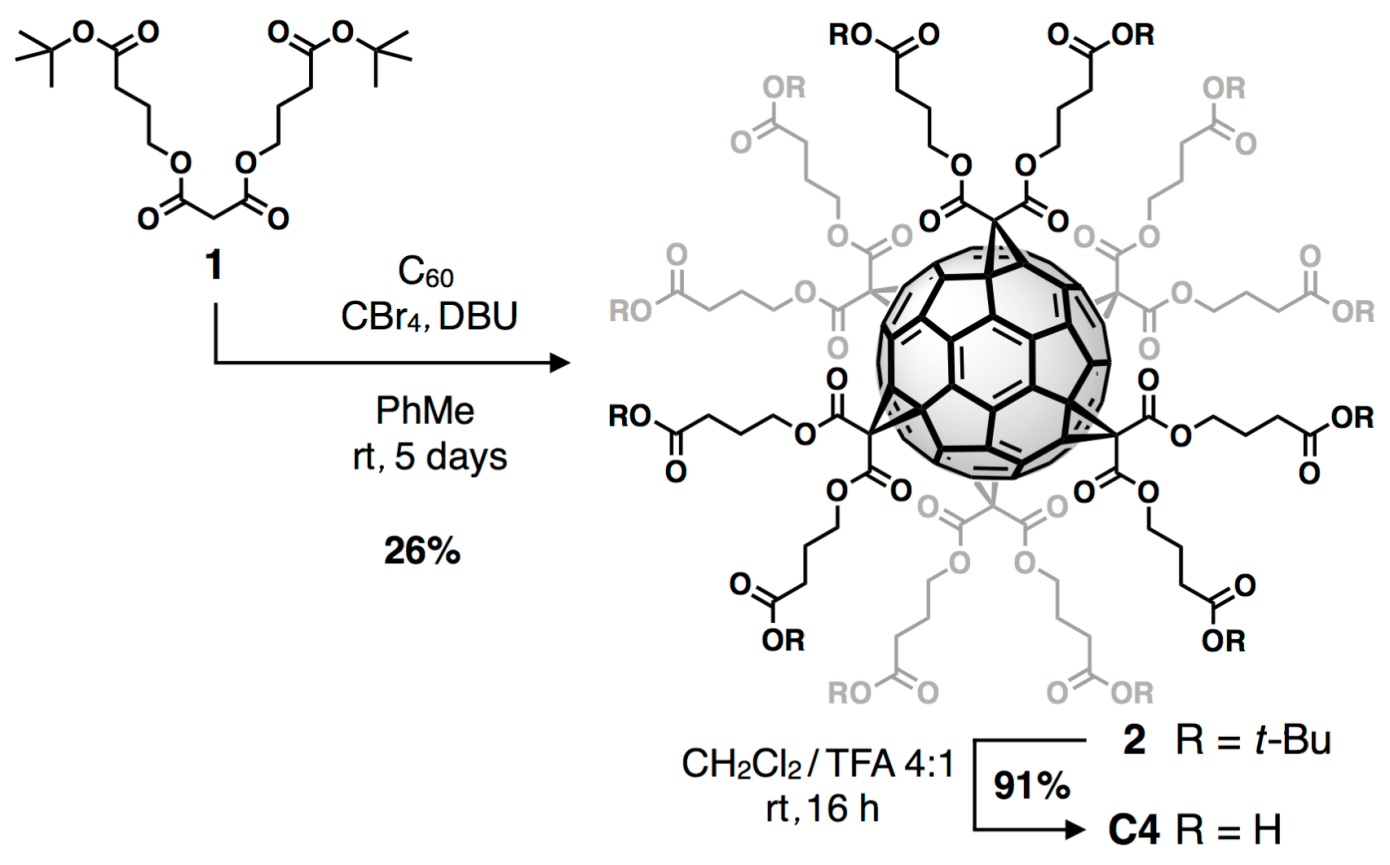
Synthesis of [60]fullerene dodecaacid **C4**.

In order to obtain porous supramolecular materials, we wondered if elongation of the alkyl spacers may result in increased fullerene–fullerene spacing associated with an enlargement of the cavitities and potential formation of a connected pore system. Therefore, we aimed for the crystallization of **C3** and also synthesized the next homologue **C4** starting from malonate **1** [59] according to a standard two-step protocol via a sixfold Bingel reaction followed by acidic deprotection (Scheme 1). As we observed the insertion of MeOH molecules into the hydrogen-bonding network of **HFF-1** [57], we also tested other polar solvents for crystallization in order to strengthen the supramolecular interactions that hold the networks together. For both **C3** and **C4**, we could finally grow single crystals suitable for X-ray diffraction by slow vapor deposition of Et_2_O into EtOH solutions of both fullerene derivatives thus resulting in the formation of frameworks **HFF-2** and **HFF-3**, respectively.

**Figure 3:**
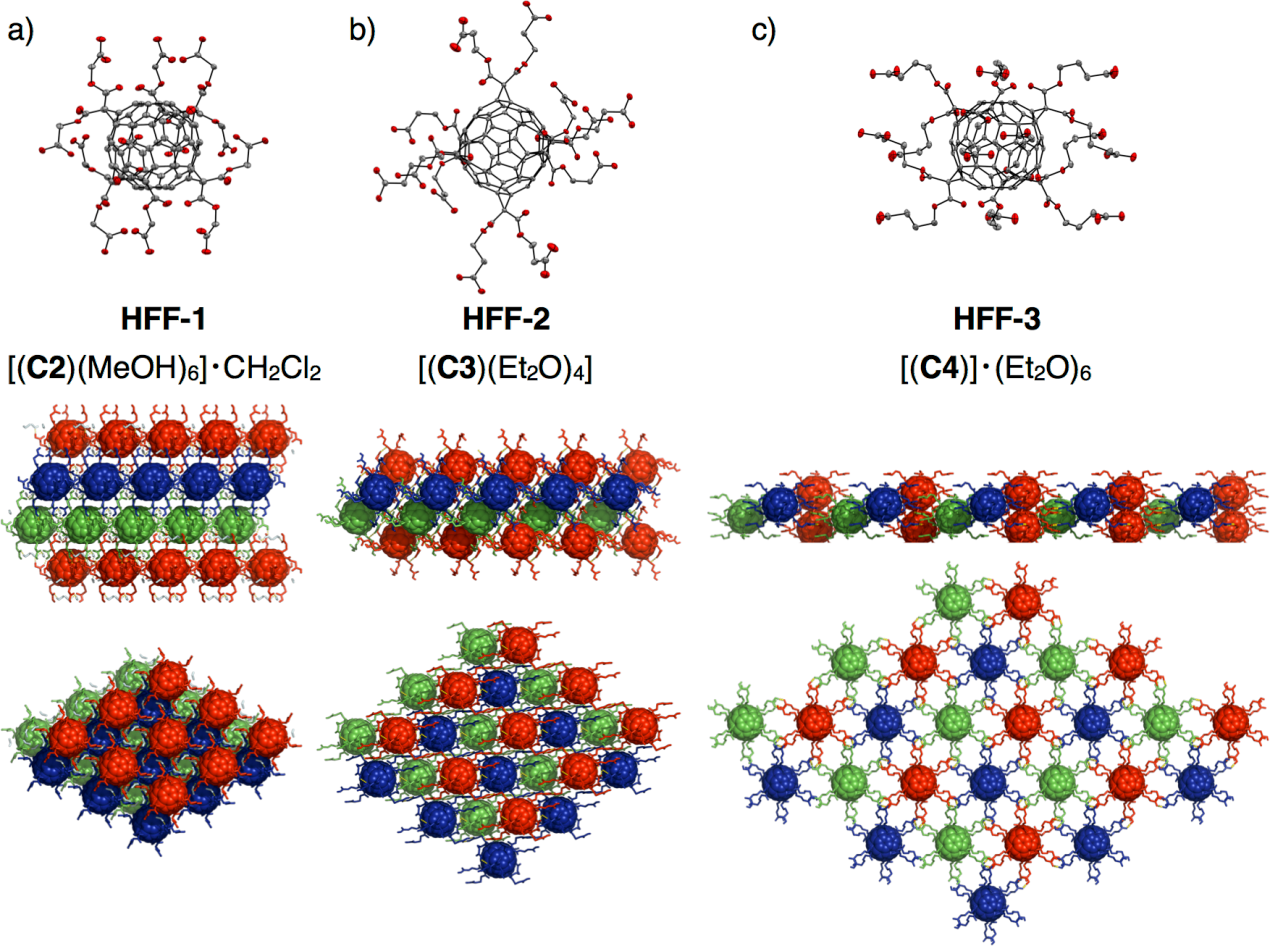
Face centered cubic arrangement of [60]fullerene dodecaacids for frameworks a) **HFF-1** [57], b) **HFF-2** and c) **HFF-3**: ORTEP representation of fullerene monomers (top, thermal ellipsoids set to 50% probability, carbon grey, oxygen red, hydrogen atoms omitted for clarity), side view indicating ABC-type packing (center) and top view indicating enlarged intralayer spacing for elongated derivatives (bottom); images are created with PyMOL [58].

**HFF-2** crystallizes in the triclinic space group 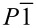 with the composition [**C3**(Et_2_O)_4_] [60]. Despite the lower symmetry compared to **HFF-1**, the packing of **HFF-2** can still be described as a fcc arrangement of **C3** molecules, which are cross-linked by hydrogen bonding. Thereby, six of the twelve side arms form linear COOH dimers (four intralayer and two interlayer) and two carboxylic acids are bound to malonate ester groups from adjacent layers (see Figure S10 in Supporting Information File 1). The remaining four carboxylic acid side chains do not participate but rather coordinate one Et_2_O molecule each, resulting in the formation of larger cavities filled with four solvent molecules (Figure 2b). Yet, these voids are still separated from each other and therefore not accessible for solvent exchange and porosity. Then again, dodecaacid **C4** possessing elongated butyric acid side chains crystallizes in the trigonal space group 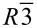 with the composition [**C4**]·6Et_2_O [61] exhibiting a flattened fcc arrangement of **C4** molecules. Figure 3 illustrates the effect of spacer elongation on the fullerene distances, thus leading to larger intralayer spacing and shorter interlayer distances with increasing length of the alkyl spacers separating the hydrogen bonding sites from the malonate units.

**Figure 4:**
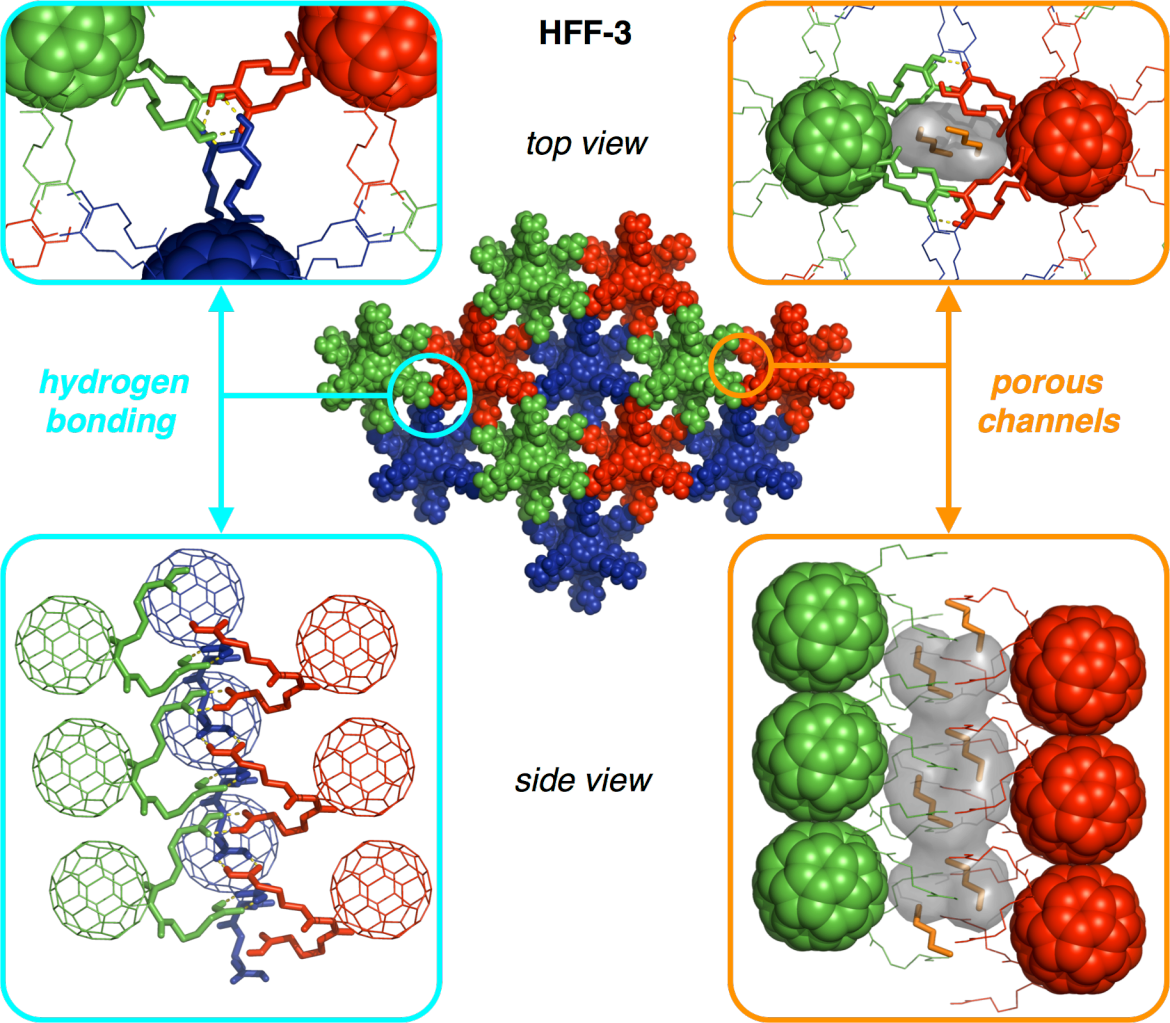
Hydrogen bonding network for **HFF-3** (left, hydrogen bonds yellow) and porous channels along the c axis filled with unbound Et_2_O molecules (right, Et_2_O orange, inner pore surface indicated in grey); images are created with PyMOL [58].

**Figure 5:**
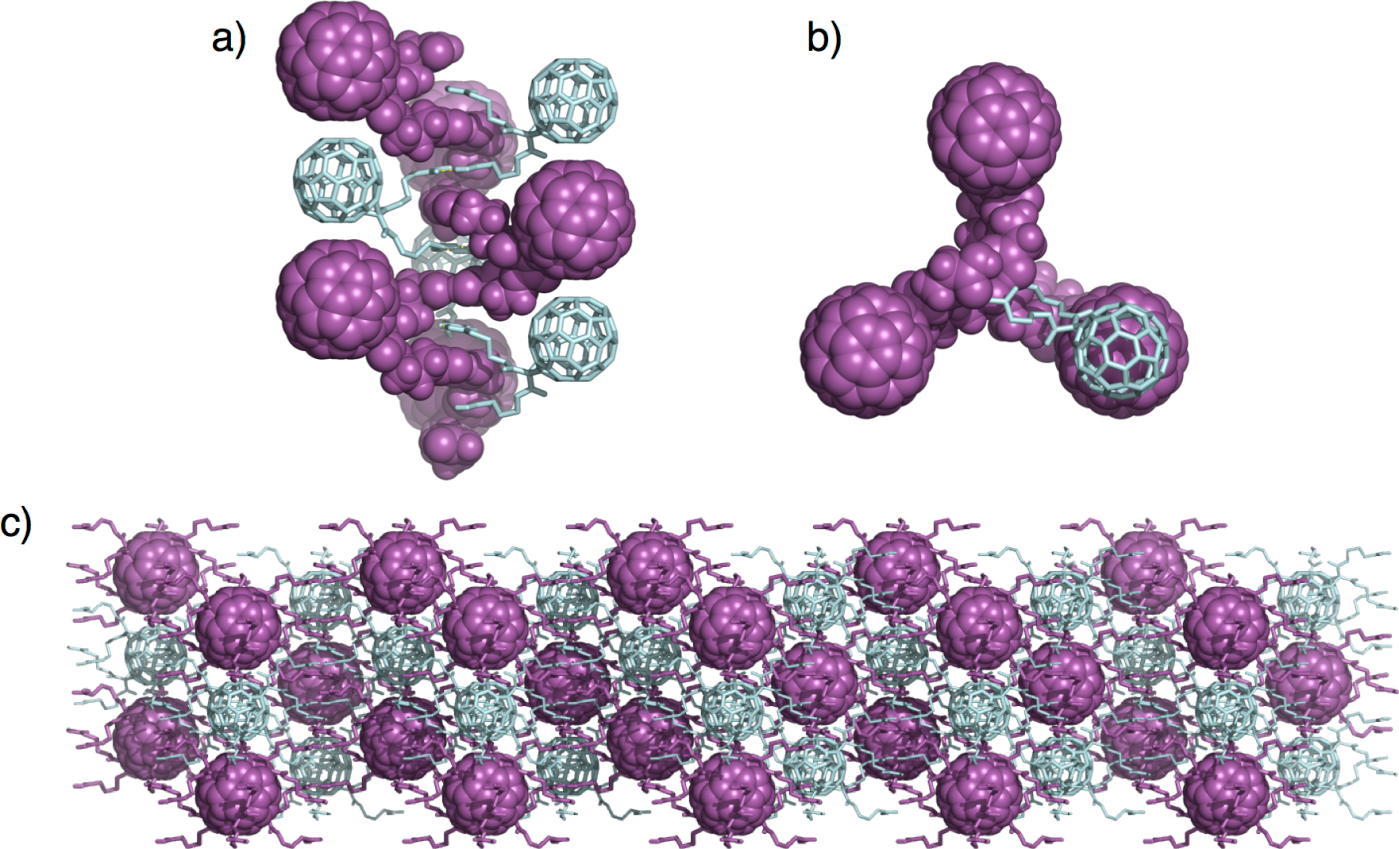
Interpenetration of two distinct hydrogen bonding networks for **HFF-3**: a) side view indicating the spiral staircase-like cross-linking of individual molecules of **C4**, b) top view and c) side view highlighting the interpenetration of two networks colored in purple and cyan.

For **HFF-3**, the butyric acid side arms are stretched out and form six pairs of carboxylic acid dimers with their closest neighbors from the next but one layers (left part of Figure 4). Interestingly, this packing motif results in the interpenetration of two independent hydrogen bonding networks (indicated in purple and cyan in Figure 5). Therefore, the individual layers are densely packed exhibiting linear columns of fullerenes in van-der-Waals distance alternating from the two interpenetrated frameworks (right part of Figure 4 and Figure 5). On the other hand, due to the large intralayer spacing and the linear stretching of the side chains, a continuous pore system is formed along the c axis (right part of Figure 4), which is filled with Et_2_O molecules that are not bound to any carboxlic acids and may therefore be removable upon activation.

In order to elaborate on the materials properties, we synthesized **HFF-3** in bulk amounts and studied the thermal stability and sorption properties of this framework. The framework crystallizes as anisotropic needles and PXRD measurements in combination with BFDH morphology calculations indicated the structural integrity of the material and the fact that the pore channels are located along the long fiber axis (see Figure S15 in Supporting Information File 1). Thermal treatment of crystalline samples under a microscope indicated partial disintegration and cracking of the crystals at elevated temperatures above 40 °C, presumably due to the removal of solvent molecules. At 198 °C, melting of the crystals occurs (see Figure S12 in the Supporting Information File 1). These findings were also confirmed by TG/DTA measurements for both as-synthesized and preactivated (evacuation at 70 °C) samples, thus showing a weight loss of up to 16% for the nonactivated material after heating to 180 °C followed by an endothermic signal indicating melting of the crystals (see Figure S17 in Supporting Information File 1). Elemental analysis of an activated sample is in perfect agreement with a solvent-free structure (see Experimental section).

In order to activate **HFF-3** and to utilize the channel system for porosity, the compound was activated for 48 hours at different stages of vacuum (10^−3^ to 10^−6^ mbar) from room temperature to 70 °C. Sorption properties were determined for N_2_ and Ar gas adsorption via a BET study at 77 K. Therein, the framework shows Henry behavior and no microporosity with a surface area of 40 m^2^ g^−1^ for N_2_ and only 18 m^2^ g^−1^ for Ar (see Figure S14 in Supporting Information File 1). However, the measured N_2_ surface area of 40 m^2^ g^−1^ may indicate that the material still retains some porosity since this value is higher than it would be anticipated for sole coverage of the outer surface of the crystals. Since the channels are aligned along the long fiber axis, kinetic effects might also hamper efficient gas uptake. As this result did not point towards accessible microchannels of the crystal structure, the material was checked for structural and chemical integrity subsequent to activation. As stated, elemental analysis fits very well, so that decomposition is unlikely. Therefore, also SEM investigations by electron microscopy were carried out on activated and non-activated samples of **HFF-3**. They corroborate the strong anisotropic character of crystalline needles of the X-ray structure determination and do not show changes upon activation (see Figure S13 in Supporting in Information File 1). However, powder X-ray diffraction indicates a change in the pattern upon activation (see Figure S16 in Supporting Information File 1). Accordingly, a yet non-identified change in the structure occurs, which may lead to the non-accessibility of the channels. One possible explanation for the observed change upon activation might be that the flexible nature of the alkyl spacers facilitates structural reorganization resulting in a potential blocking of the channels after removal of the solvent molecules. The Et_2_O molecules appear to play a crucial role for the stabilization of both channel size and shape. For future investigations, the implementation of fullerene derivatives possessing long but rigid spacer units might be beneficial for retaining porosity of such supramolecular crystals.

## Conclusion

We have presented the crystallization of two [60]fullerene dodecaacids possessing three-dimensional hydrogen bonding networks **HFF-2** and **HFF-3** in the solid state. Different extensions of the linker arms have been investigated for newly synthesized **C4** and compared to the known **C2** and **C3** lengths. Depending on the length of the linker arms, the distance of the fullerene moieties in the framework compounds increases resulting in a new framework structure and giving rise to the idea of permanent porosity for larger fullerene separation. **HFF-3** shows channels suitable for microporosity. However, during the activation process not only release of the solvent molecules from the channels but also a structural change occurs that leads to a Henry behavior in BET investigations. In order to rigidify the structures and stabilize the pore systems upon solvent removal, novel fullerene derivatives possessing less flexible spacers need to be designed and synthesized. Efforts in this regard are currently in progress in our laboratories.

## Experimental

Hexakisadduct **C3** [55] and malonate **1** [59] were synthesized according to literature procedures. X-ray crystallography: Bruker D8 Quest diffractometer with Photon 100 CMOS APS detector and Montel multilayer optics monochromated Cu *K*_α_ radiation. PXRD diffraction: Bruker D8 Discovery with 1D-Lynxeye detector using Cu *K*_α_ radiation (unsplit *K*_α1_ + *K*_α2_ doublet, mean wavelength λ = 154.19pm), reflection and transmission geometry.

**Hexakisadduct 2**: C_60_ (565 mg, 785 µmol, 1 equiv), malonate **1** (3.10 g, 7.84 mmol, 10 equiv) and CBr_4_ (26.0 g, 78.4 mmol, 100 equiv) were dissolved in dry toluene (500 mL). DBU (2.34 mL, 15.7 mmol, 20 equiv, 60 mL solution in dry toluene) was added dropwise within 20 minutes resulting in a color change from purple to dark-red. After additional stirring for five days at room temperature, the mixture was passed through a short silica-pad with ethyl acetate as eluent in order to remove the solvent and traces of unreacted C_60_. After further column chromatographic separation (SiO_2_; toluene/ethyl acetate 10:1), pure hexakisadduct **2** (600 mg, 197 µmol, 25%) was obtained as a yellow crystalline solid. mp >200 °C dec; ^1^H NMR (400 MHz, CDCl_3_, rt) δ 1.44 (s, 108H, C(C*H_3_*)_3_), 1.99 (m, ^3^*J* = 6.8 Hz, 24H, CH_2_C*H_2_*CH_2_), 2.31 (t, ^3^*J* = 7.4 Hz, 24H, C*H_2_*CO_2_* t*-Bu), 4.30 ppm (t, ^3^*J* = 6.52 Hz, 24H, *CH_2_*CH_2_CO_2_*t*-Bu); ^13^C NMR (100 MHz, CDCl_3_, rt) δ 24.10 (12C, CH_2_*C*H_2_CH_2_), 28.26 (36C, C(*C*H_3_)_3_), 31.80 (12C, *C*H_2_CO_2_* t*-Bu), 45.25 (6C, O_2_C*C*H_2_CO_2_), 66.17 (12C, *C*H_2_CH_2_CO_2_* t*-Bu), 69.16 (12C, C_60_ sp^3^), 80.72 (12C, *C*(CH_3_)_3_), 141.15 (24C, C_60_ sp^2^), 145.99 (24C, C_60_ sp^2^), 163.75 (12C, O_2_CCH_2_*C*O_2_), 171.89 ppm (12C, *C*O_2_* t*-Bu); UV–vis (CH_2_Cl_2_) λ: 281, 315 (sh), 334 (sh) nm; MS (MALDI, DCTB, pos) *m/z*: 3038 [M]^+^; anal, calcd for C_174_H_180_O_48_: C, 68.76; H, 5.97; found: C, 68.87; H, 6.09.

**Hexakisadduct C4**: TFA (1.7 mL) was added to a solution of **2** (250 mg, 86.3 µmol) in CH_2_Cl_2_ (7 mL). The mixture was stirred for one day at room temperature. The precipitated yellow solid was isolated through decantation. Further purification was carried out by suspending the raw product in CH_2_Cl_2_, centrifugation and collection through decantation. After drying under high vacuum, hexakisadduct **C4** (185 mg, 78.2 µmol, 91%) was obtained as a light-yellow solid. mp 198 °C; ^1^H NMR (400 MHz, DMSO-*d*_6_, rt) δ 1.85 (m, 24H, CH_2_C*H*_2_CH_2_), 2.27 (t, 24H, C*H*_2_CO_2_H), 4.30 (t, 24H, COO*CH*_2_), 12.16 ppm (s br, 12H, CO_2_*H*); ^13^C NMR (100 MHz, DMSO-*d*_6_, rt) δ 23.47 (12C, *C*H_2_CO_2_H), 29.71 (12C, CH_2_*C*H_2_CH_2_), 45.44 (6C, O_2_C*C*H_2_CO_2_), 66.35 (12C, COO*C*H_2_), 68.67 (12C, C_60_ sp^3^), 140.71 (24C, C_60_ sp^2^), 145.02 (24C, C_60_ sp^2^), 162.75 (12C, O_2_CCH_2_*C*O_2_), 173.64 ppm (12C, *C*O_2_H); UV–vis (CH_2_Cl_2_) λ: 281, 315 (sh), 334 (sh) nm; MS (MALDI, DCTB, pos) *m/z*: 2365 [M]^+^; anal. calcd for C_126_H_84_O_48_: C, 63.96; H, 3.58; found: C, 63.96; H, 3.58.

**Crystal growth of HFF-2**: Single crystals suitable for X-ray diffraction have been obtained by slow vapor deposition of Et_2_O into a solution of **C3** in EtOH. CCDC deposition number: 1498265.

**Crystal growth of HFF-3**: Single crystals suitable for X-ray diffraction have been obtained by slow vapor deposition of Et_2_O into a solution of **C4** in EtOH. CCDC deposition number: 1498266.

## Supporting Information

File 1 Analytical and crystallographic data; SEM, BET, PXRD and TGA/DTA data.

## References

[1] Slater A G, Cooper A I (2015). Science.

[2] Morris R E, Wheatley P S (2008). Angew Chem, Int Ed.

[3] Mastalerz M (2012). Chem – Eur J.

[4] Corma A, García H, Llabrés i Xamena F X (2010). Chem Rev.

[5] Catti L, Zhang Q, Tiefenbacher K (2016). Chem – Eur J.

[6] Allendorf M D, Bauer C A, Bhakta R K, Houk R J T (2009). Chem Soc Rev.

[7] Chen Y, Ma S (2012). Rev Inorg Chem.

[8] Heine J, Müller-Buschbaum K (2013). Chem Soc Rev.

[9] Meyer L V, Schönfeld F, Müller-Buschbaum K (2014). Chem Commun.

[10] Liu D, Lu K, Poon C, Lin W (2014). Inorg Chem.

[11] Hu Z, Deibert B J, Li J (2014). Chem Soc Rev.

[12] Roy S, Chakraborty A, Maji T K (2014). Coord Chem Rev.

[13] Müller-Buschbaum K, Beuerle F, Feldmann C (2015). Microporous Mesoporous Mater.

[14] Wang C, Zhang T, Lin W (2012). Chem Rev.

[15] Rybak J-C, Hailmann M, Matthes P R, Zurawski A, Nitsch J, Steffen A, Heck J G, Feldmann C, Götzendörfer S, Meinhardt J (2013). J Am Chem Soc.

[16] Dogru M, Bein T (2014). Chem Commun.

[17] Kitagawa S, Kitaura R, Noro S (2004). Angew Chem, Int Ed.

[18] Férey G (2008). Chem Soc Rev.

[19] Feng X, Ding X, Jiang D (2012). Chem Soc Rev.

[20] Ding S-Y, Wang W (2013). Chem Soc Rev.

[21] Zhang G, Mastalerz M (2014). Chem Soc Rev.

[22] Hasell T, Cooper A I (2016). Nat Rev Mater.

[23] Mastalerz M, Schneider M W, Oppel I M, Presly O (2011). Angew Chem, Int Ed.

[24] Schneider M W, Oppel I M, Ott H, Lechner L G, Hauswald H-J, Stoll R, Mastalerz M (2012). Chem – Eur J.

[25] Brutschy M, Schneider M W, Mastalerz M, Waldvogel S R (2012). Adv Mater.

[26] Schneider M W, Oppel I M, Griffin A, Mastalerz M (2013). Angew Chem, Int Ed.

[27] Zhang G, Presly O, White F, Oppel I M, Mastalerz M (2014). Angew Chem, Int Ed.

[28] Elbert S M, Rominger F, Mastalerz M (2014). Chem – Eur J.

[29] Lim S, Kim H, Selvapalam N, Kim K-J, Cho S J, Seo G, Kim K (2008). Angew Chem, Int Ed.

[30] Comotti A, Bracco S, Distefano G, Sozzani P (2009). Chem Commun.

[31] Yang W, Greenaway A, Lin X, Matsuda R, Blake A J, Wilson C, Lewis W, Hubberstey P, Kitagawa S, Champness N R (2010). J Am Chem Soc.

[32] He Y, Xiang S, Chen B (2011). J Am Chem Soc.

[33] Dalapati S, Saha R, Jana S, Patra A K, Bhaumik A, Kumar S, Guchhait N (2012). Angew Chem, Int Ed.

[34] Mastalerz M, Oppel I M (2012). Angew Chem.

[35] Luo X-Z, Jia X-J, Deng J-H, Zhong J-L, Liu H-J, Wang K-J, Zhong D-C (2013). J Am Chem Soc.

[36] Natarajan R, Bridgland L, Sirikulkajorn A, Lee J-H, Haddow M F, Magro G, Ali B, Narayanan S, Strickland P, Charmant J P H (2013). J Am Chem Soc.

[37] Chen T-H, Popov I, Kaveevivitchai W, Chuang Y-C, Chen Y-S, Daugulis O, Jacobson A J, Miljanić O S (2014). Nat Commun.

[38] Li P, He Y, Arman H D, Krishna R, Wang H, Weng L, Chen B (2014). Chem Commun.

[39] Li P, He Y, Guang J, Weng L, Zhao J C-G, Xiang S, Chen B (2014). J Am Chem Soc.

[40] Lü J, Perez-Krap C, Suyetin M, Alsmail N H, Yan Y, Yang S, Lewis W, Bichoutskaia E, Tang C C, Blake A J (2014). J Am Chem Soc.

[41] Hisaki I, Nakagawa S, Tohnai N, Miyata M (2015). Angew Chem, Int Ed.

[42] Li P, He Y, Zhao Y, Weng L, Wang H, Krishna R, Wu H, Zhou W, O'Keeffe M, Han Y (2015). Angew Chem, Int Ed.

[43] Wang H, Li B, Wu H, Hu T-L, Yao Z, Zhou W, Xiang S, Chen B (2015). J Am Chem Soc.

[44] Patil R S, Banerjee D, Zhang C, Thallapally P K, Atwood J L (2016). Angew Chem, Int Ed.

[45] Zhou D-D, Xu Y-T, Lin R-B, Mo Z-W, Zhang W-X, Zhang J-P (2016). Chem Commun.

[46] Kohl B, Rominger F, Mastalerz M (2014). Org Lett.

[47] Yan W, Seifermann S M, Pierrat P, Bräse S (2015). Org Biomol Chem.

[48] Beuerle F, Hirsch A (2009). Chem – Eur J.

[49] Nierengarten J-F, Iehl J, Oerthel V, Holler M, Illescas B M, Muñoz A, Martín N, Rojo J, Sánchez-Navarro M, Cecioni S (2010). Chem Commun.

[50] Dey S K, Beuerle F, Olson M A, Stoddart J F (2011). Chem Commun.

[51] Hörmann F, Hirsch A (2013). Chem – Eur J.

[52] Luczkowiak J, Muñoz A, Sánchez-Navarro M, Ribeiro-Viana R, Ginieis A, Illescas B M, Martín N, Delgado R, Rojo J (2013). Biomacromolecules.

[53] Muñoz A, Sigwalt D, Illescas B M, Luczkowiak J, Rodríguez-Pérez L, Nierengarten I, Holler M, Remy J-S, Buffet K, Vincent S P (2016). Nat Chem.

[54] Peng P, Li F-F, Neti V S P K, Metta-Magana A J, Echegoyen L (2014). Angew Chem, Int Ed.

[55] Kraft A, Roth P, Schmidt D, Stangl J, Müller-Buschbaum K, Beuerle F (2016). Chem – Eur J.

[56] Kraft A, Beuerle F (2016). Tetrahedron Lett.

[57] Kraft A, Gsänger M, Beuerle F (2014). Eur J Org Chem.

[58] The PyMOL Molecular Graphics System.

[59] Witte P, Hörmann F, Hirsch A (2009). Chem – Eur J.

[R60] 60Supplementary crystallographic data for **HFF-2** can be obtained free of charge from The Cambridge Crystallographic Data Centre via http://www.ccdc.cam.ac.uk/data_request/cif (CCDC 1498265); crystal data: C_114_H_60_O_48_·4C_4_H_10_O, *M* = 2494.09 g mol^−1^, triclimic, 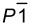 , *a* = 13.1513(5), *b* = 14.4631(7), *c* = 15.2117(7) Å, α = 107.332(3), β = 95.752(3)°, γ = 94.594(2)°, *V* = 2729.6(2) Å3, *Z* = 1, ρ_calc_ = 1.517 g cm^−3^, µ(Cu *K*_α_) = 1.010 mm^−1^, *T* = 100(2) K; 42237 independent measured reflections. *F*2 refinement, *R*_1_ = 0.0490, w*R*_2_ = 0.1302 (observed), 11068 independent observed reflections (*R*_int_ = 0.0346) [|F_0_| > 4σ(|F_0_|), 2Θ ≤ 149.4°], 830 parameters, no restraints.

[R61] 61Supplementary crystallographic data for **HFF-3** can be obtained free of charge from The Cambridge Crystallographic Data Centre via http://www.ccdc.cam.ac.uk/data_request/cif (CCDC 1498266); crystal data: C_126_H_84_O_48_·6C_4_H_10_O, *M* = 2810.64 g mol^−1^, trigonal, 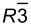 , *a* = 33.8114(9), *b* = 33.8114(9), *c* = 9.8056(3) Å, α = 90, β = 90, γ = 120°, *V* = 9708.0(6) Å3, *Z* = 3, ρ_calc_ = 1.442 g cm^−3^, µ(Cu *K*_α_) = 0.927 mm^−1^, *T* = 100(2) K; 44836 independent measured reflections. *F*2 refinement, *R*_1_ = 0.0488, w*R*_2_ = 0.1414 (observed), 4438 independent observed reflections (*R*_int_ = 0.0442) [|F_0_| > 4σ(|F_0_|), 2Θ ≤ 150.28°], 350 parameters, no restraints.

