# Supplementary material for: Supramolecular frameworks based on [60]fullerene hexakisadducts
Source: Beilstein J Org Chem. 2017 Jan 2;13:1–9. doi: 10.3762/bjoc.13.1 (PMC5238557; doi:10.3762/bjoc.13.1)
Supplement: File 1 — Analytical and crystallographic data; SEM, BET, PXRD and TGA/DTA data. [file Beilstein_J_Org_Chem-13-01-s001.pdf]

Supporting Information

for

Supramolecular frameworks based on [60]fullerene hexakisadducts

Andreas Kraft[1], Johannes Stangl[2], Ana-Maria Krause[1], Klaus Müller-Buschbaum[2]

and Florian Beuerle[1]*§

Address: [1]Institut für Organische Chemie & Center for Nanosystems Chemistry, Universität Würzburg, Am Hubland, 97074 Würzburg, Germany and [2]Institut für Anorganische Chemie, Universität Würzburg, Am Hubland, 97074 Würzburg, Germany

Email: Florian Beuerle - florian.beuerle@uni-wuerzburg.de

§Tel.: +49 931 31-83603

*Corresponding author

Analytical and crystallographic data; SEM, BET, PXRD and TGA/DTA data

Content

1 Analytical data ____ S2

2 Crystallographic data ____ S6

3 Electron microscopy (SEM) ____ S12

4 Sorption study (BET) ____ S13

5 Powder X-ray diffraction (PXRD) ____ S14

6 TGA/DTA study ____ S16

1 Analytical data

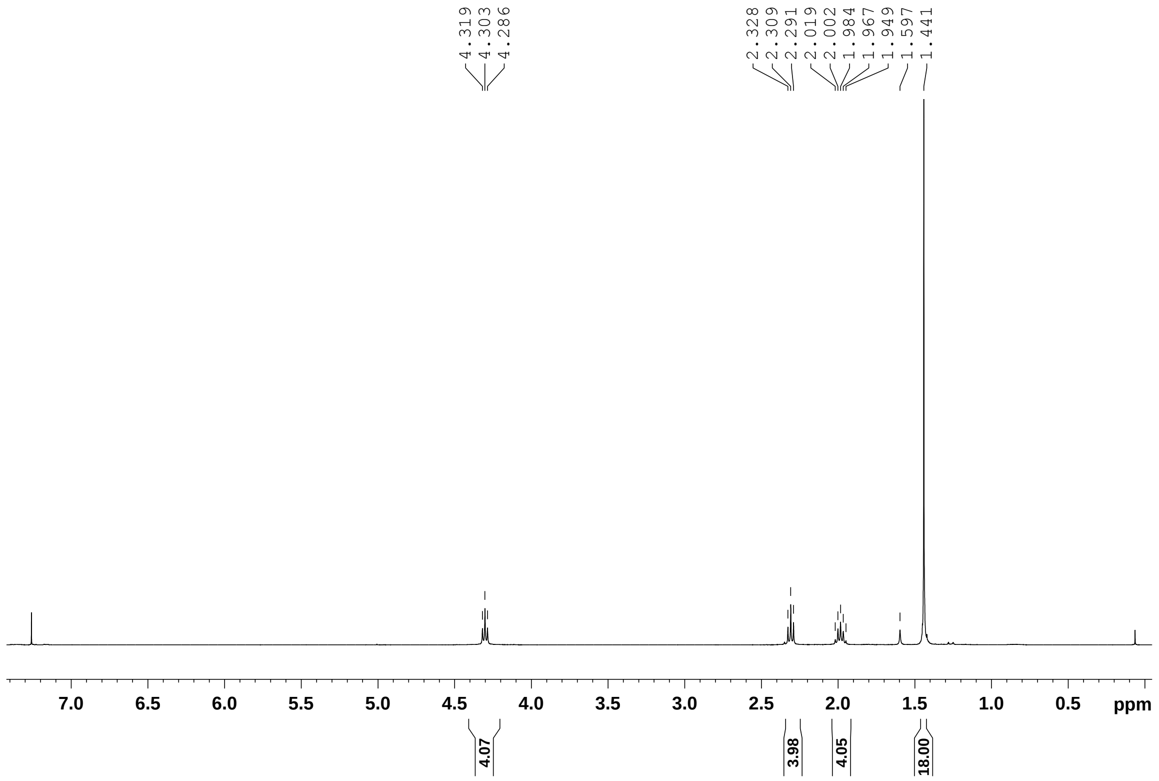

Figure S1: ^{1}H NMR (400 MHz, $CDCl_3$, rt) for hexakisadduct **2**.

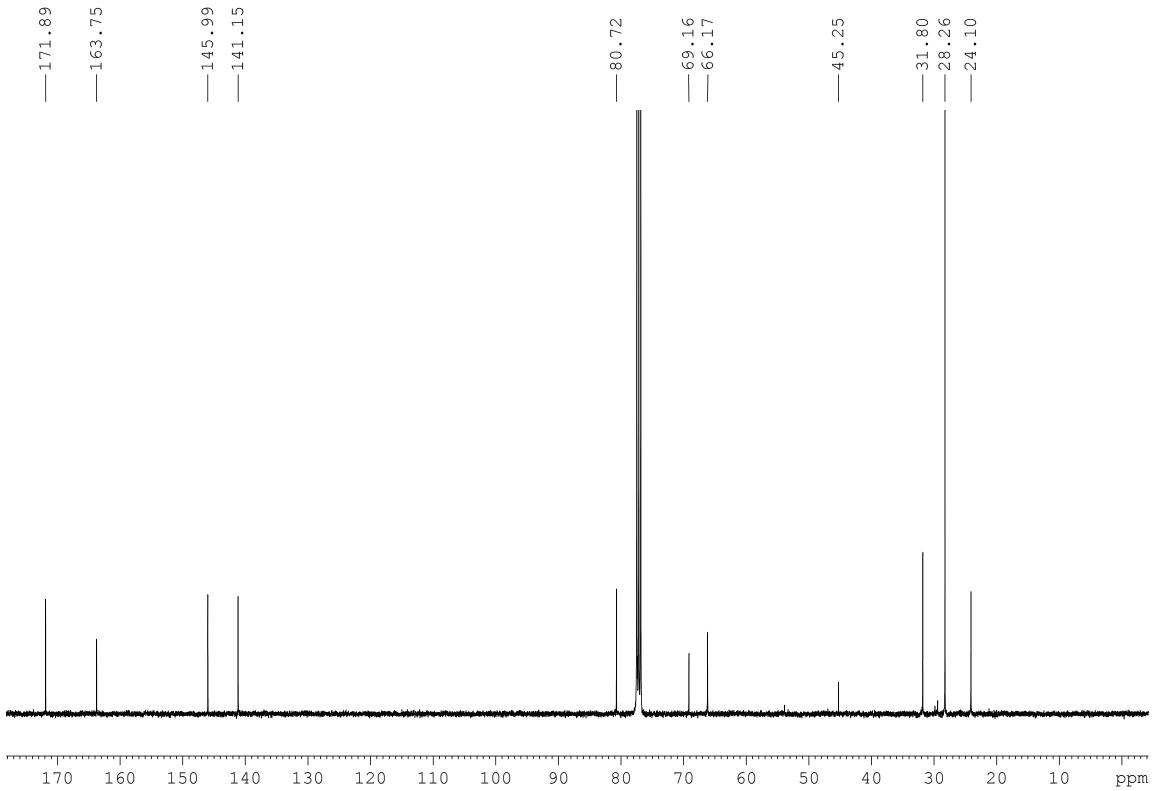

Figure S2: ^{13}C NMR (100 MHz, $CDCl_3$, rt) for hexakisadduct **2**.

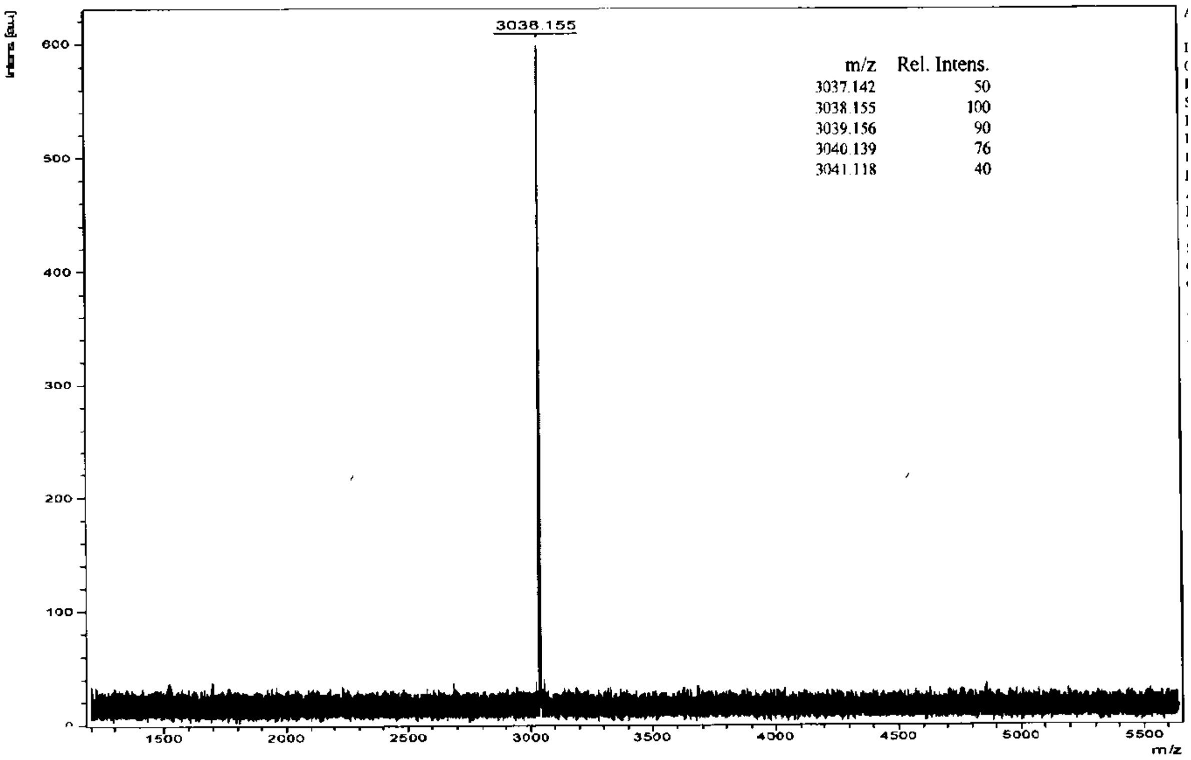

Figure S3: MS (MALDI, DCTB, pos) for hexakisadduct **2**.

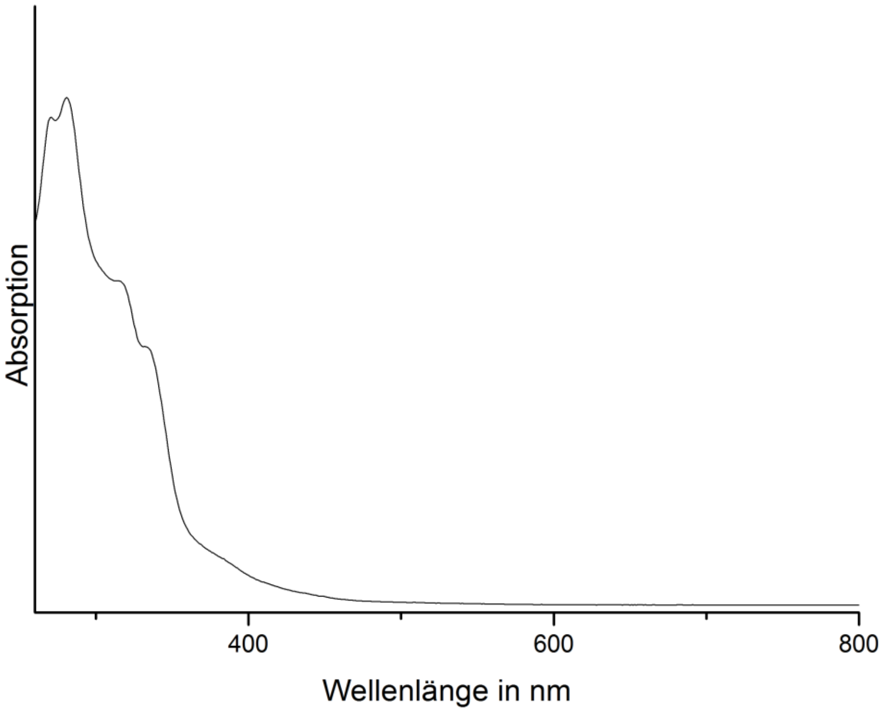

Figure S4: UV–vis (CH_2Cl_2, rt) for hexakisadduct **2**.

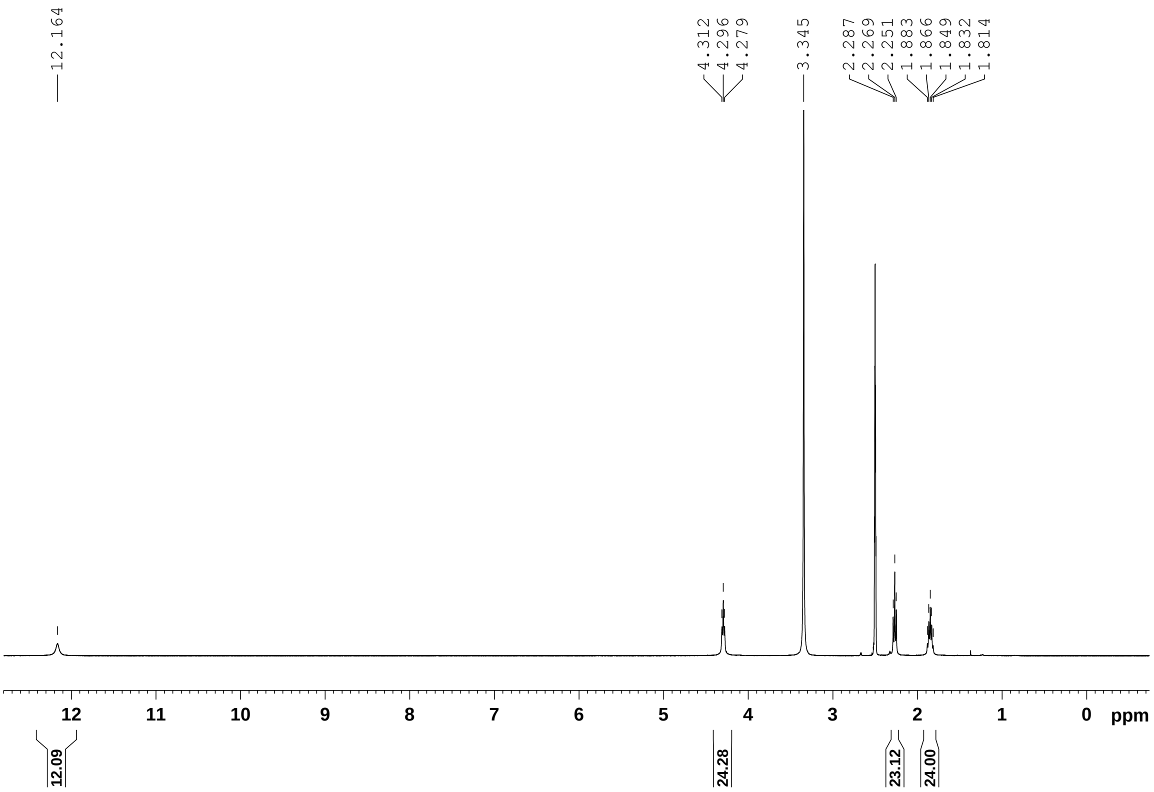

Figure S5: ^{1}H NMR (400 MHz, DMSO-d_6, rt) for hexakisadduct **C4**.

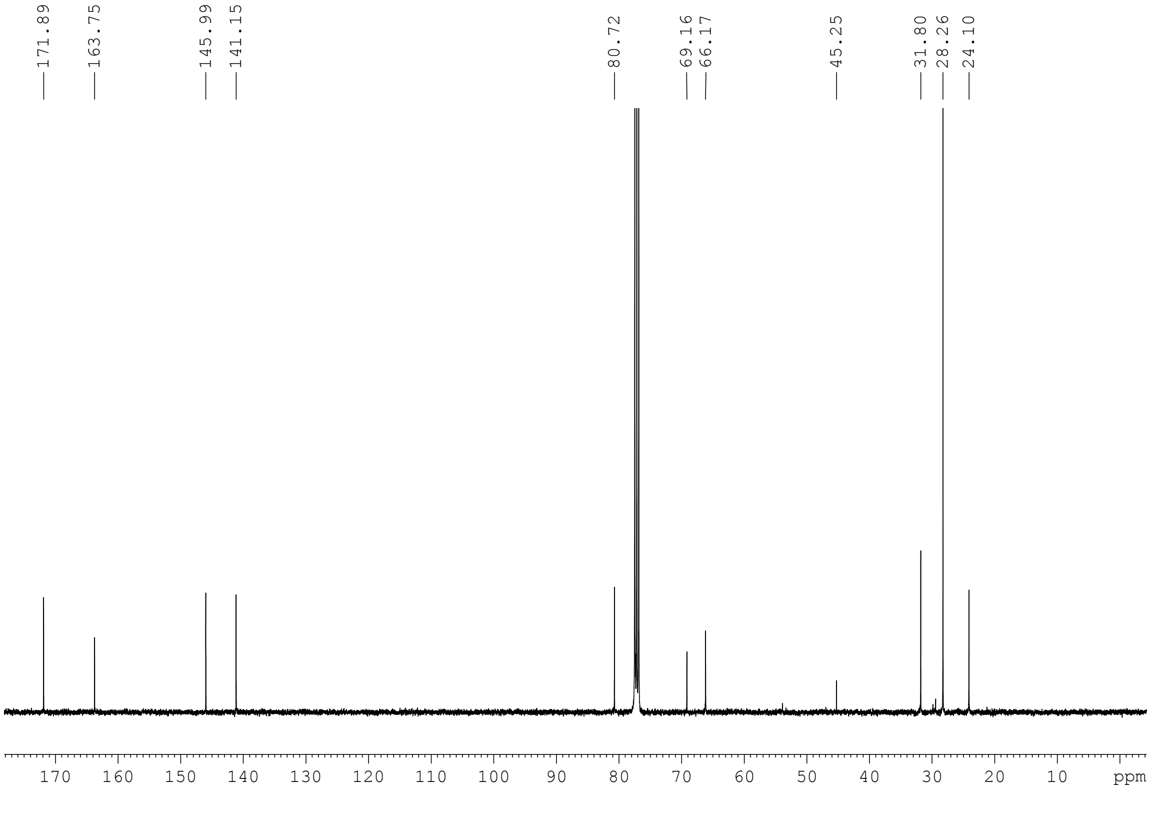

Figure S6: ^{13}C NMR (100 MHz, DMSO-d_6, rt) for hexakisadduct **C4**.

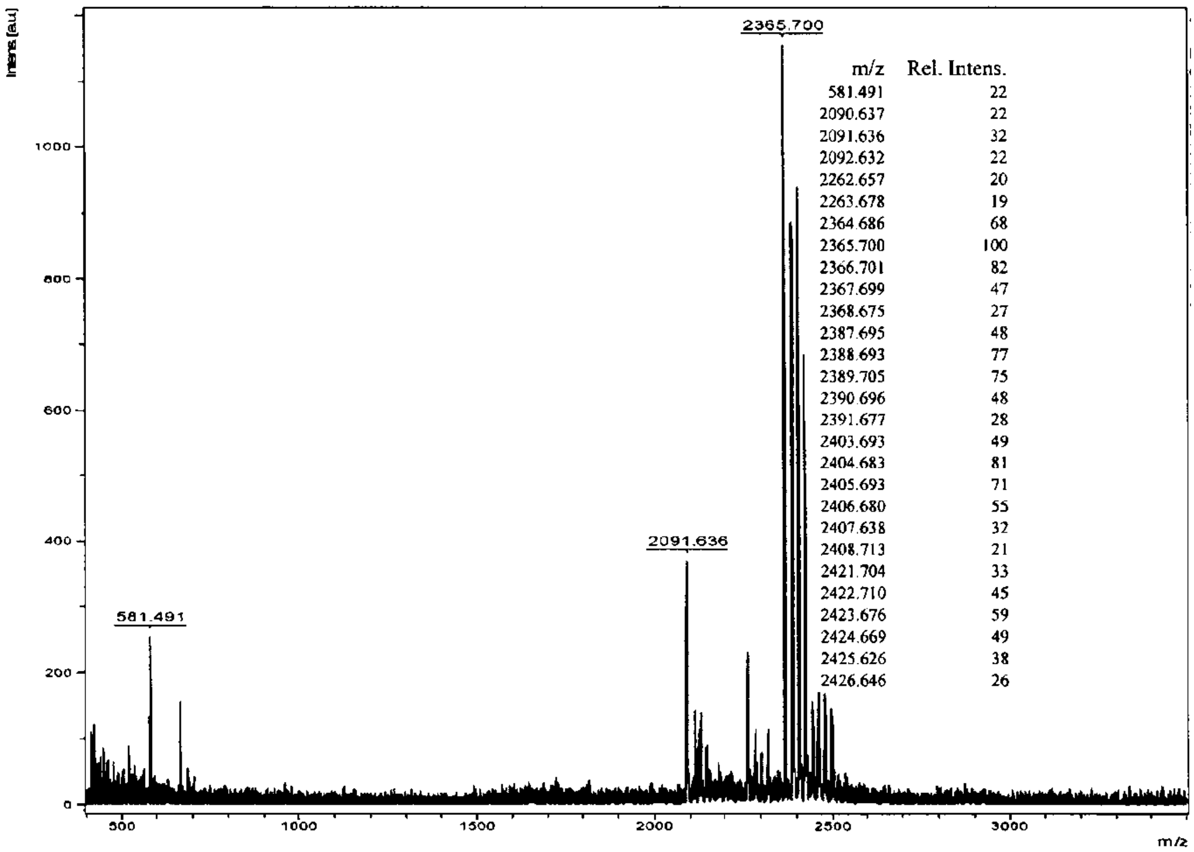

Figure S7: MS (MALDI, DCTB, pos) for hexakisadduct **C4**.

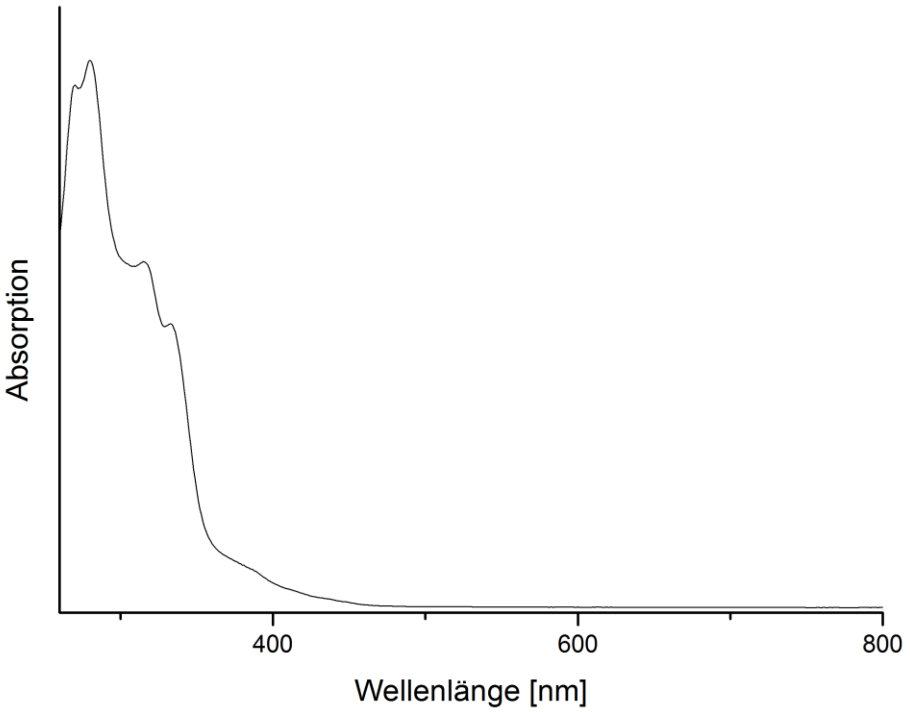

Figure S8: UV–vis (MeOH, rt) for hexakisadduct **C4**.

2 Crystallographic data

Single crystals were mounted on a 100 µm MiTeGen MicroLoop using perfluorinated polyalkylether. Single crystal X-ray diffraction data were collected on a Bruker D8 Quest Kappa diffractometer with a Photon100 CMOS detector and multi-layered mirror monochromated CuK_{α} radiation. The images were processed with the Bruker software packages and equivalent reflections were merged. The data were corrected for absorption effects using semi-empirical methods from equivalents. Corrections for Lorentz and polarization effects were applied. The structures were solved by direct methods, refined with the SHELXTL software package (G. Sheldrick, *Acta Crystallogr. Sect. A*, 2008, **64**, 112) and expanded using Fourier techniques. All non-hydrogen atoms were refined anisotropically. Hydrogen atoms were assigned to geometrically idealized positions and were included in structure factor calculations.

Crystal data and refinement details for HFF-2:

Suitable crystals for X-ray diffraction have been obtained by slow vapor deposition of Et_2O into a solution of **C3** in EtOH. Supplementary crystallographic data for **HFF-2** can be obtained free of charge from The Cambridge Crystallographic Data Centre via www.ccdc.cam.ac.uk/data_request/cif (CCDC 1498265).

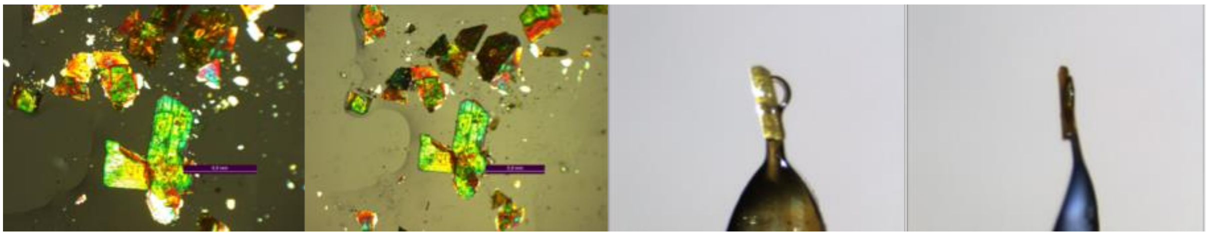

Crystal data and refinement details for HFF-3:

Suitable crystals for X-ray diffraction have been obtained by slow vapor deposition of Et_2O into a solution of **C4** in EtOH. Et_2O molecules in the pores are disordered (main site occupancy 57%). Supplementary crystallographic data for **HFF-3** can be obtained free of charge from The Cambridge Crystallographic Data Centre via www.ccdc.cam.ac.uk/data_request/cif (CCDC 1498266).

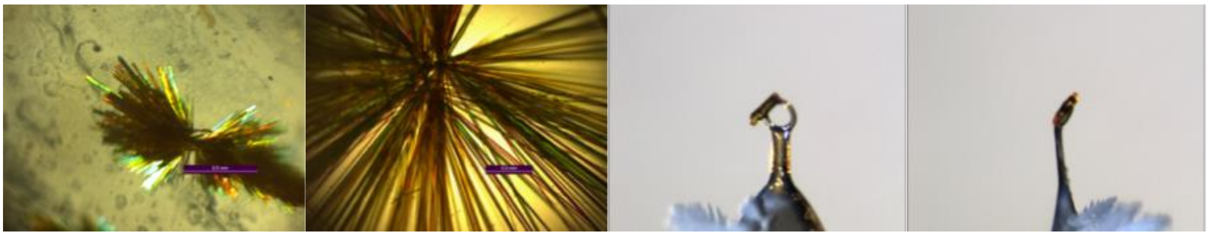

Table S1: Crystal data and structure refinement for **HFF-2**.

Empirical formula	$C_{114}H_{60}O_{48} \cdot 4\ C_4H_{10}O$
Formula weight	2494.09 g mol^{-1}
Temperature	100(2) K
Wavelength	1.54178 Å
Crystal system, space group	Triclinic, $P\bar{1}$ (Nr. 2)
Unit cell dimensions	a = 13.1513(5) Å, α = 107.332(3)°<br>b = 14.4631(7) Å, β = 95.752(3)°<br>c = 15.2117(7) Å, γ = 94.594(2)°
Volume	2729.6(2) Å^3
Z, calculated density	1, 1.517 g cm^{-1}
Absorption coefficient	1.010 mm^{-1}
F(000)	1296
Crystal size	0.453 x 0.044 x 0.028 mm^3
Θ range for data collection	3.069 to 74.700°
Limiting indices	$-16 \leq h \leq 12$, $-18 \leq k \leq 18$, $-19 \leq l \leq 19$
Reflections collected / unique	42237 / 11068 [R_{int} = 0.0346]
Completeness to Θ = 67.679	99.6%
Absorption correction	Semi-empirical from equivalents
Max. and min. transmission	0.7538 and 0.6426
Refinement method	Full-matrix least-squares on F^2
Data / restraints / parameters	11068 / 0 / 830
Goodness-of-fit on F^2	1.040
Final R indices [$I > 2(\sigma(I)$]	R_1 = 0.0490, wR_2 = 0.1302
R indices (all data)	R_1 = 0.0622, wR_2 = 0.1398
Largest diff. peak and hole	0.561 and −0.584 e Å^3

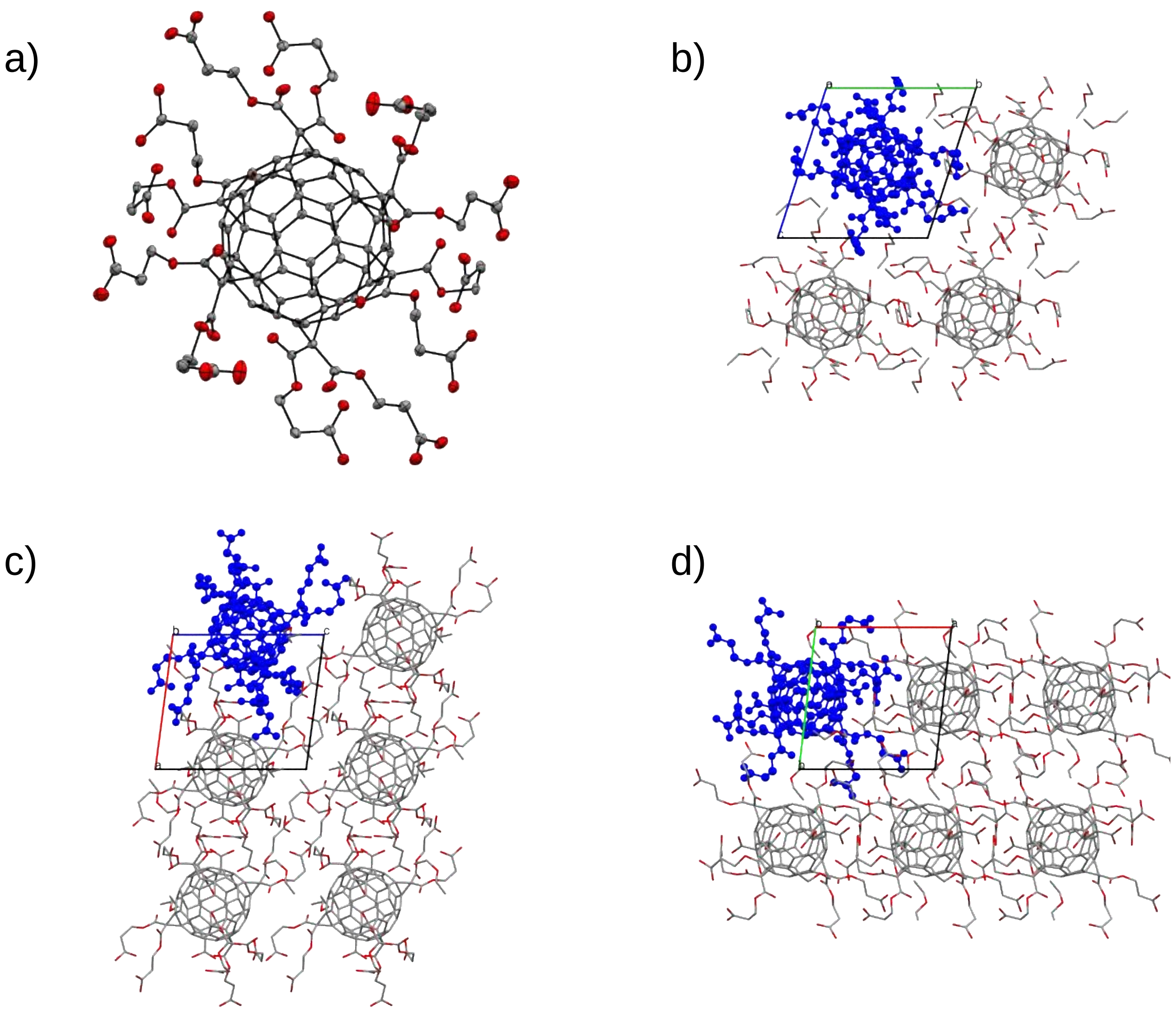

Figure S9: Single-crystal X-ray structure of **HFF-2**: a) ORTEP representation of single molecule of **C3** (thermal ellipsoids set to 50% probability, carbon grey, oxygen red, hydrogen atoms omitted for clarity); crystal packing with views along the crystallographic b) a, c) b and d) c axis (single molecule of **C3** is highlighted in blue).

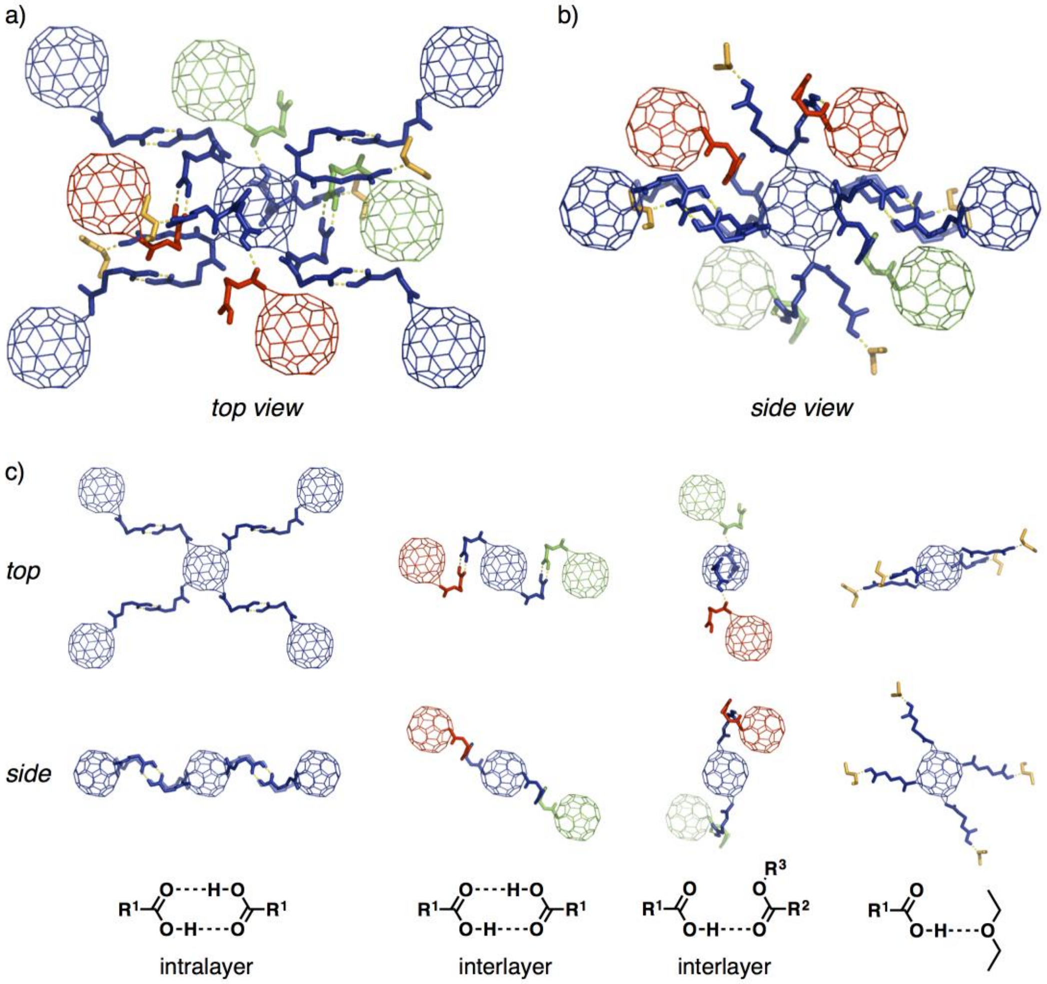

Figure S10: Hydrogen bonding motifs for **HFF-2**: a) top and b) side view of hydrogen bonding interactions for one molecule of **C3**; c) different types of hydrogen bonds observed in **HFF-2**; *color code: layer A red, layer B blue, layer C green, Et_2O orange, hydrogen bonds yellow; non-participating side arms and non polar hydrogen atoms omitted for clarity*.

Table S2: Crystal data and structure refinement for **HFF-3**.

Empirical formula	$C_{126}H_{84}O_{48} \cdot 6\ C_4H_{10}O$
Formula weight	2810.64 g mol^{-1}
Temperature	100(2) K
Wavelength	1.54178 Å
Crystal system, space group	Trigonal, $R\bar{3}$ (Nr. 148)
Unit cell dimensions	a = 33.8114(9) Å, α = 90°<br>b = 33.8114(9) Å, β = 90°<br>c = 9.8056(3) Å, γ = 120°
Volume	9708.0(6) $Å^3$
Z, calculated density	3, 1.442 g cm^{-1}
Absorption coefficient	0.927 mm^{-1}
F(000)	4428
Crystal size	0.194 x 0.059 x 0.032 mm^3
Θ range for data collection	2.614 to 75.142°
Limiting indices	−40 ≤ h ≤ 42, −42 ≤ k ≤ 42, −12 ≤ l ≤ 12
Reflections collected / unique	44836 / 4438 [R_{int} = 0.0442]
Completeness to Θ = 67.679	100%
Absorption correction	Semi-empirical from equivalents
Max. and min. transmission	0.7538 and 0.6709
Refinement method	Full-matrix least-squares on F^2
Data / restraints / parameters	4438 / 0 / 350
Goodness-of-fit on F^2	1.066
Final R indices [$I > 2(\sigma(I)$]	R_1 = 0.0488, wR_2 = 0.1414
R indices (all data)	R_1 = 0.0556, wR_2 = 0.1471
Largest diff. peak and hole	0.419 and −0.424 e $Å^3$

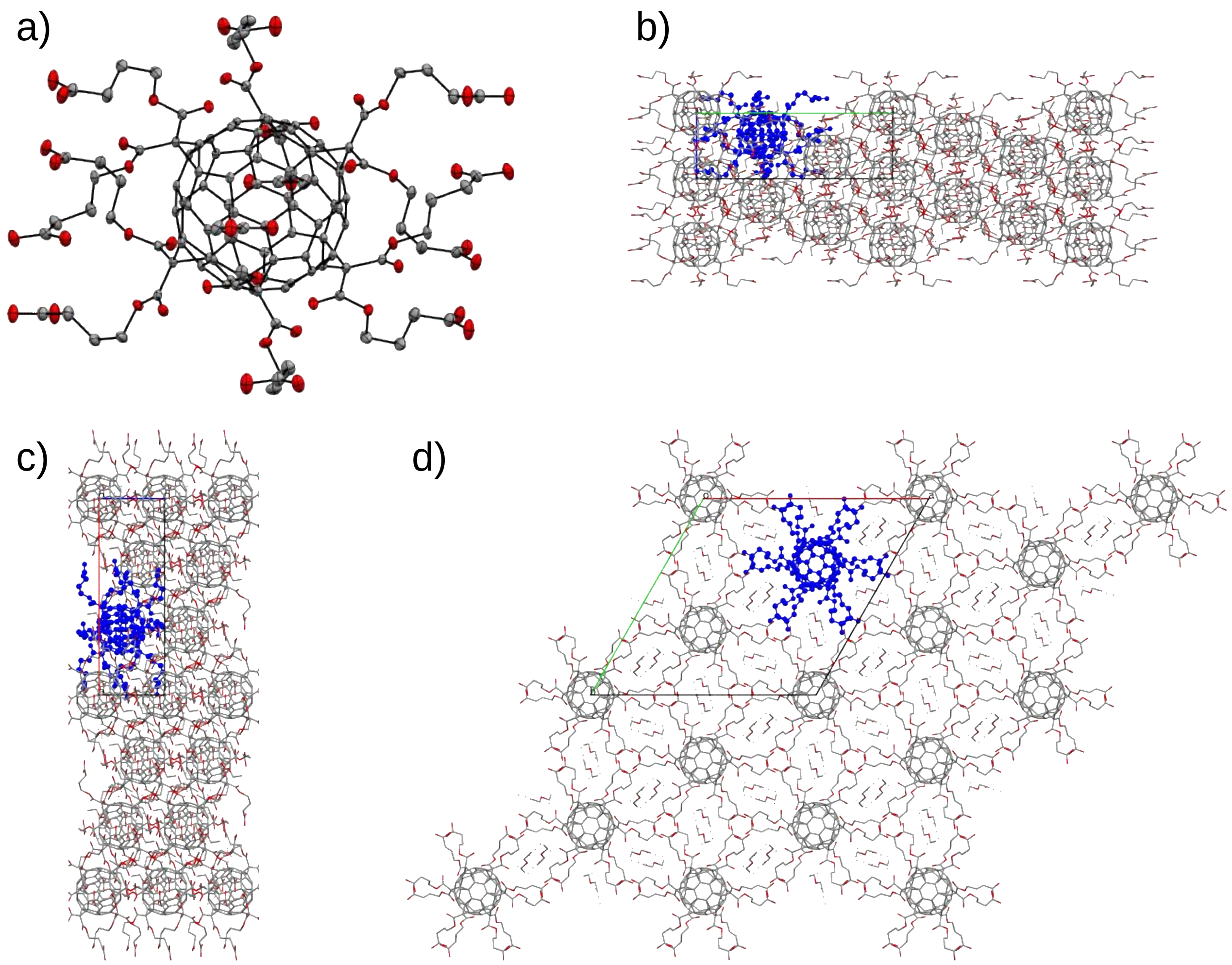

Figure S11: Single-crystal X-ray structure of **HFF-3**: a) ORTEP representation of single molecule of **C4** (thermal ellipsoids set to 50% probability, carbon grey, oxygen red, hydrogen atoms omitted for clarity); crystal packing with views along the crystallographic b) a, c) b and d) c axis (single molecule of **C4** is highlighted in blue).

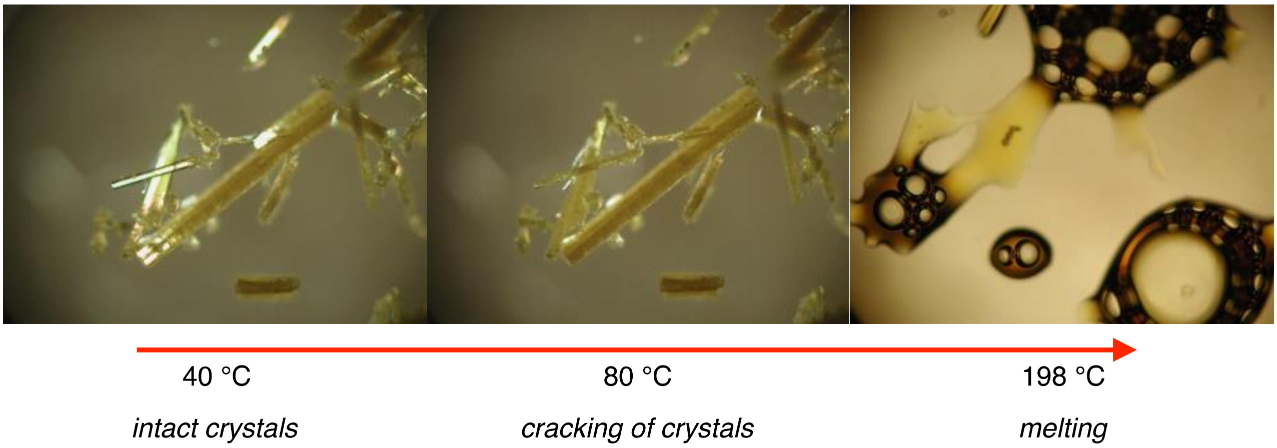

Figure S12: Thermal behavior of crystalline **HFF-3** under ambient conditions.

3 Electron microscopy (SEM)

Morphological investigations were determined on a field emission scanning electron microscope (FE-SEM) ULTRA plus (Zeiss) with a GEMINI e-beam column at 5 kV.

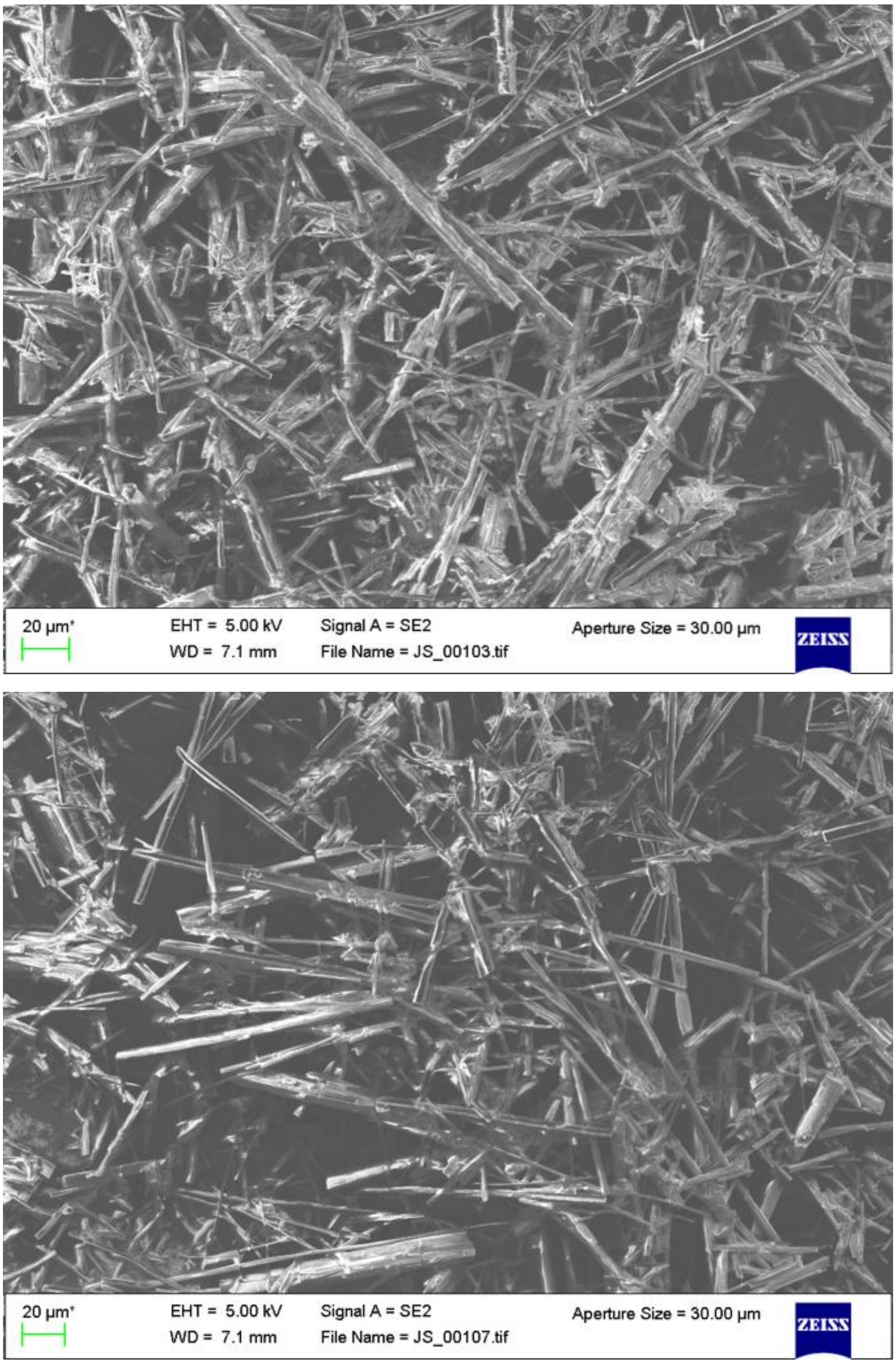

Figure S13: SEM images of **HFF-3** as-synthesized (top) and subsequent to activation (bottom) indicating the strong anisotropic crystal habitus, which does not change upon activation.

4 Sorption study (BET)

All sorption experiments were carried out on a Quantachrome Autosorb AS-1C. N_2 (Linde Gas, purity > 99.999%) and Ar (Linde Gas, purity > 99.999%) physisorption were determined at 77 K with dynamic p_0-determination via a p0-cell at p = 760 mmHg. Analysis and interpretation of data were done with the Quantachrome AS1Win software package, version 2.11. HFF-3 samples were activated at room temperature and at 70 °C with pressures of 2.0×10^{-3} mbar and 1×10^{-6} mbar for 24 h, each. All samples were treated in the outgas station, until outgassing rates were at least below 2 microns/minute in pressure increase and afterwards loaded with He (Linde Gas, purity > 99.999%) before analysis was carried out.

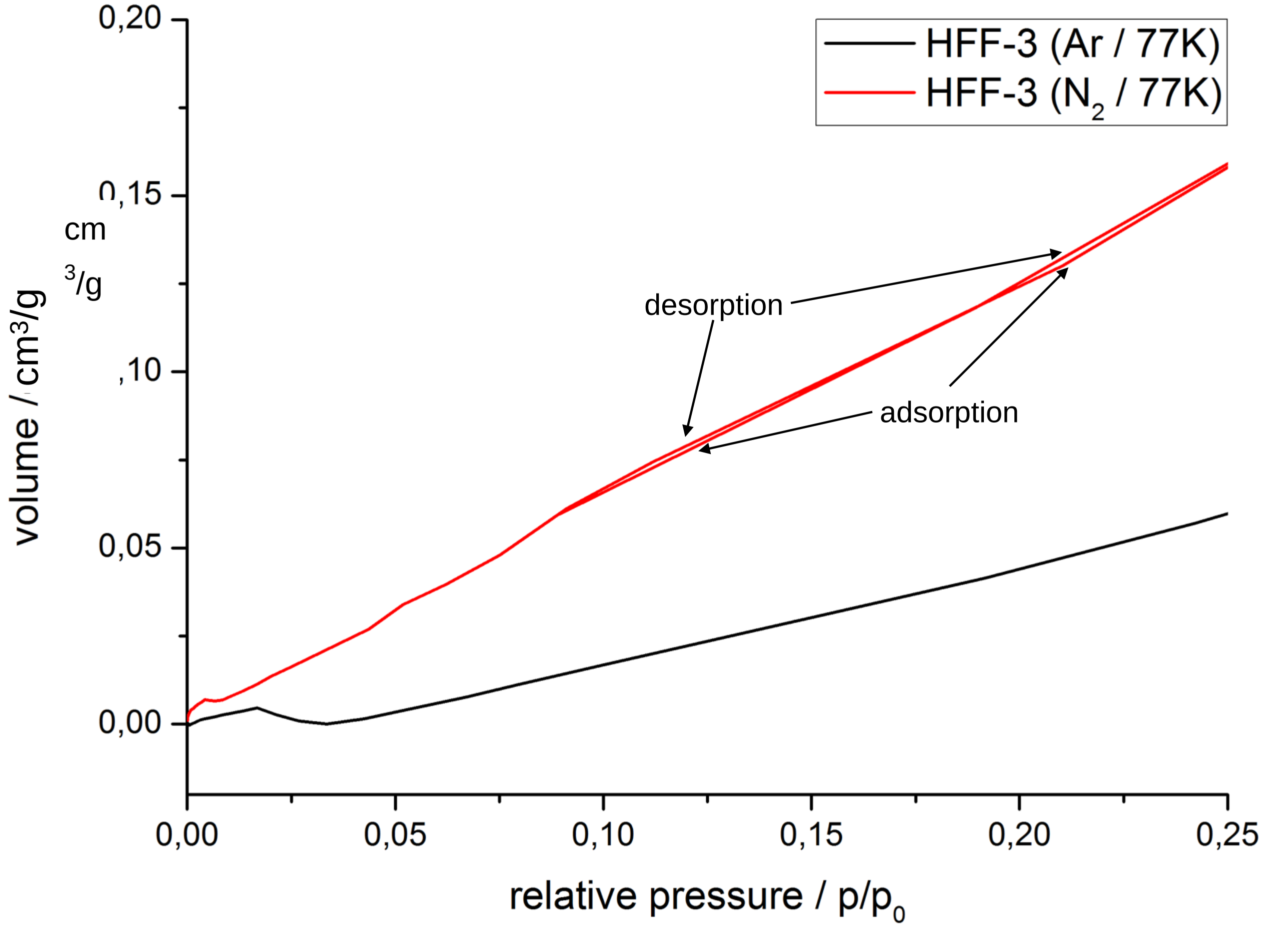

Figure S14: Adsorption and desorption isotherms of **HFF-3** for N_2 (red) and Ar adsorption (black) at 77K.

Measured BET surface areas for **HFF-3**:	40 $m^2\ g^{-1}$ for N_2 and 18 $m^2\ g^{-1}$ for Ar.	
solvent accessible pore volume:	394 $Å^3$/unit cell (4.1%)	0.033 $cm^3\ g^{-1}$
solvent accessible surface area:	1130 $Å^2$/unit cell	958 $m^2\ g^{-1}$

Theoretical values calculated with Materials Studio (*Dassault Systèmes BIOVIA, Materials Studios 2016, San Diego: Dassault Systèmes, 2016)* from the crystal structure without Et_2O molecules.

5 Powder X-ray diffraction (PXRD)

Powder diffraction samples were either grinded and put into Lindemann glass capillaries (Ø 0.3 mm) for the as-synthesized product or investigated on a plate with dome sample holder for the activated samples. Diffraction data was collected on a BRUKER AXS D8 Discover powder X-ray diffractometer equipped with Lynx-Eye detector in transmission geometry (as-synthesized) and reflection geometry (activated). The X-ray radiation (Cu-Kα1; λ = 154.06 pm) was focused with a Goebel mirror, Cu-Kα2 radiation was eliminated by the application of a Ni absorber. Diffraction patterns were recorded and analysed using the program package BRUKER AXS Diffrac-Suite.

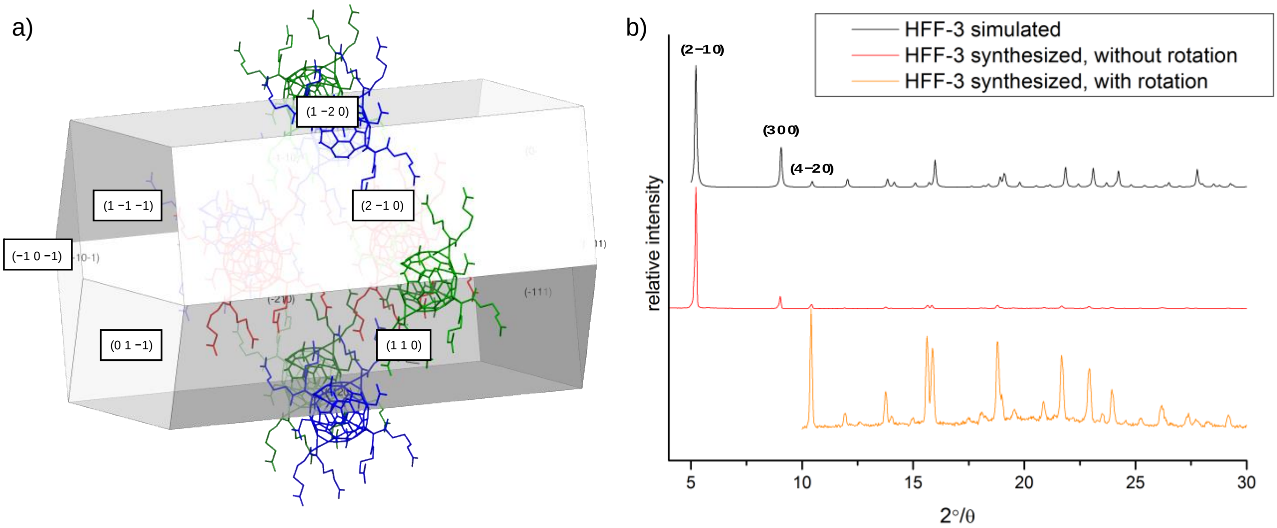

Figure S15: a) BFDH morphology as calculated from the crystal structure of **HFF-3**; b) diffraction pattern of **HFF-3** as synthesized (red: measured without rotation, orange: measured without rotation) in comparison with a pattern simulated from single-crystal data (black).

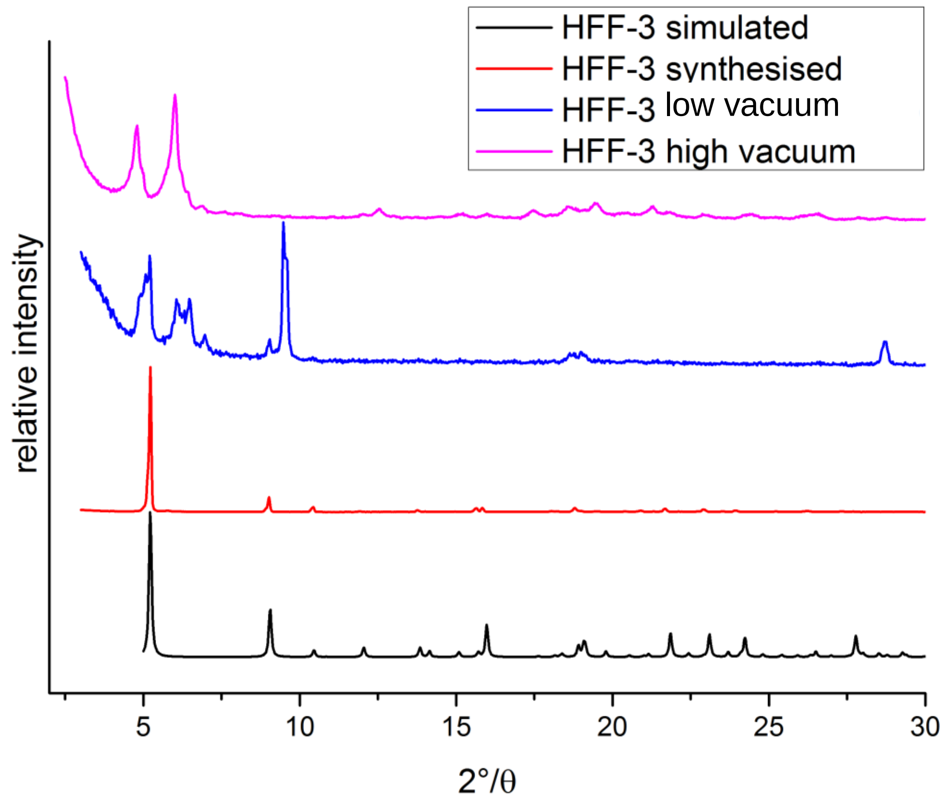

Figure S16: Diffraction patterns of **HFF-3** as-synthesized (red) in comparison with a pattern simulated with singly crystal data (black) and subsequent to activation treatment at 2×10^{-3} mbar (blue) and 1×10^{-6} mbar (pink).

6 TG/DTA study

a)

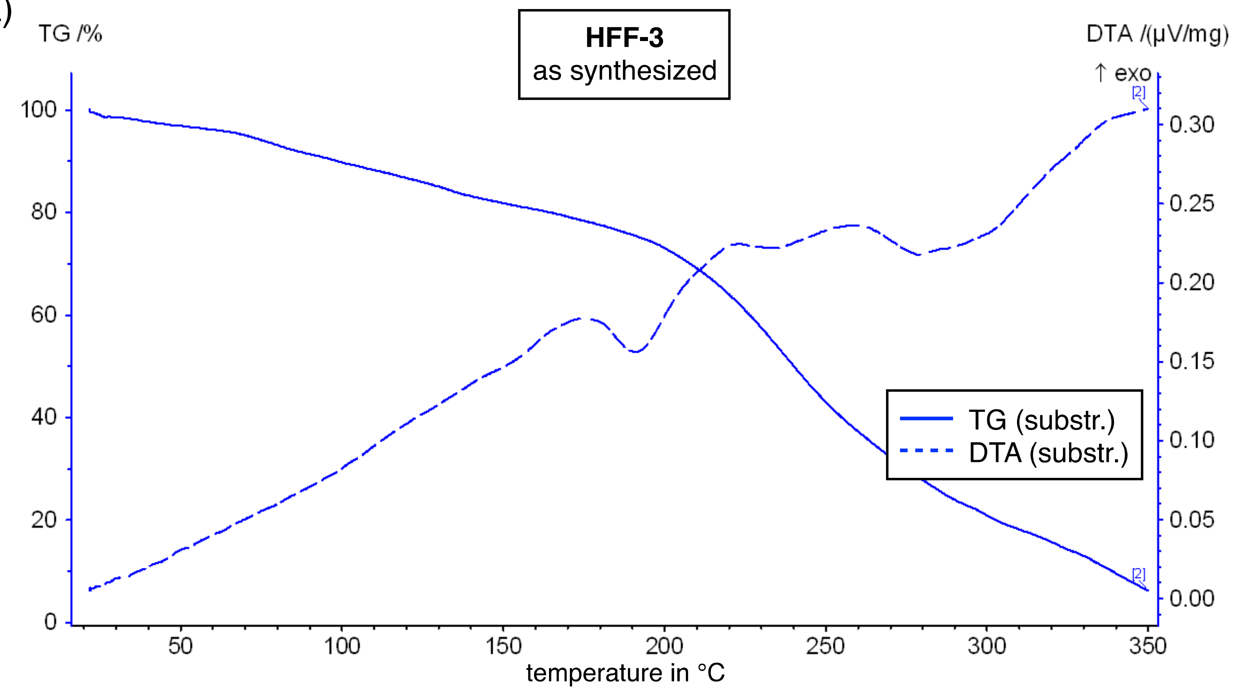

b)

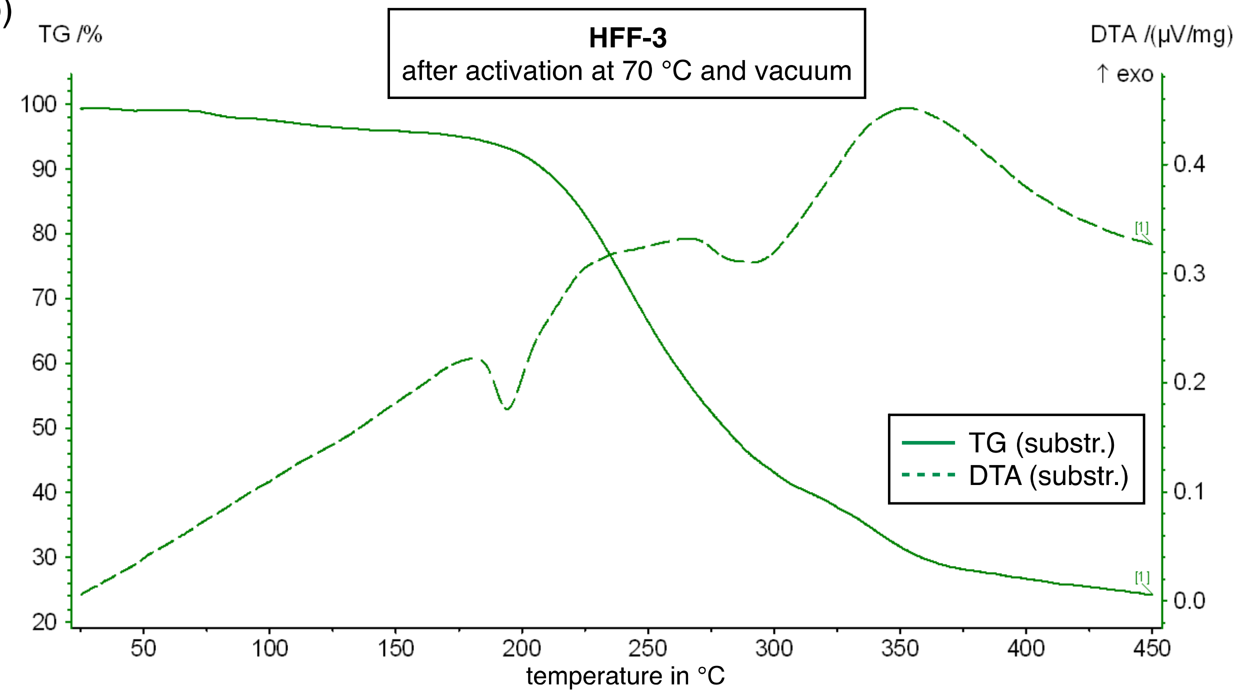

Figure S17: TG/DTA study for **HFF-3** a) as synthesized and b) after activation at 70 °C under vacuum.
